# Whole-body iron transport and metabolism: Mechanistic, multi-scale model to improve treatment of anemia in chronic kidney disease

**DOI:** 10.1371/journal.pcbi.1006060

**Published:** 2018-04-16

**Authors:** Joydeep Sarkar, Alka A. Potdar, Gerald M. Saidel

**Affiliations:** 1 Pricewaterhouse Coopers LLP, New York, NY, United States of America; 2 Department of Biomedical Engineering, Case Western Reserve University, Cleveland, OH, United States of America; 3 Department of Biomedical Engineering, Case Western Reserve University, Cleveland, OH, United States of America; University of Michigan, UNITED STATES

## Abstract

Iron plays vital roles in the human body including enzymatic processes, oxygen-transport via hemoglobin and immune response. Iron metabolism is characterized by ~95% recycling and minor replenishment through diet. Anemia of chronic kidney disease (CKD) is characterized by a lack of synthesis of erythropoietin leading to reduced red blood cell (RBC) formation and aberrant iron recycling. Treatment of CKD anemia aims to normalize RBC count and serum hemoglobin. Clinically, the various fluxes of iron transport and accumulation are not measured so that changes during disease (e.g., CKD) and treatment are unknown. Unwanted iron accumulation in patients is known to lead to adverse effects. Current whole-body models lack the mechanistic details of iron transport related to RBC maturation, transferrin (Tf and TfR) dynamics and assume passive iron efflux from macrophages. Hence, they are not predictive of whole-body iron dynamics and cannot be used to design individualized patient treatment. For prediction, we developed a mechanistic, multi-scale computational model of whole-body iron metabolism incorporating four compartments containing major pools of iron and RBC generation process. The model accounts for multiple forms of iron *in vivo*, mechanisms involved in iron uptake and release and their regulation. Furthermore, the model is interfaced with drug pharmacokinetics to allow simulation of treatment dynamics. We calibrated our model with experimental and clinical data from peer-reviewed literature to reliably simulate CKD anemia and the effects of current treatment involving combination of epoietin-alpha and iron dextran. This *in silico* whole-body model of iron metabolism predicts that a year of treatment can potentially lead to 90% downregulation of ferroportin (FPN) levels, 15-fold increase in iron stores with only a 20% increase in iron flux from the reticulo-endothelial system (RES). Model simulations quantified unmeasured iron fluxes, previously unknown effects of treatment on FPN-level and iron stores in the RES. This mechanistic whole-body model can be the basis for future studies that incorporate iron metabolism together with related clinical experiments. Such an approach could pave the way for development of effective personalized treatment of CKD anemia.

## Author summary

Iron is mostly recycled with very little loss or replenishment. This model simulates the complex regulatory network that maintains iron within healthy limits. Iron diseases are typically characterized by breakdown of such regulatory pathways (e.g. synthesis of erythropoietin, inhibition of iron release by hepcidin, etc.) that lead to pathological lack of iron or deposition of iron. Most iron metabolism research focuses on the specific roles of the regulatory proteins (e.g. HFE, ceruloplasmin), but here we chose to focus on the interplay between ferroportin and hepcidin. The current literature lacks an integrated whole-body view of iron metabolism with key fluxes that are essential for investigating the roles of regulatory protein within feedback networks and molecular pathways. These aspects can be investigated by simulations with our top-down, mechanistic computational model. Our model simulations suggest possible improvements in treatment of anemia of chronic kidney disease. Furthermore, this model can provide a platform for future developments of powerful predictive tools that can be used to accelerate drug development of iron-disorder diseases.

## Introduction

Iron is essential for a wide variety of biological functions. Its most critical physiological function is associated with the oxygen carrying capacity of hemoglobin. Iron also plays important roles in mitochondrial redox reactions (cytochromes) and in healthy immune function [1, 2]. While there is a large focus on the multitude of diseases implicated due to iron deficiency, excessive iron is also very toxic [1]. Hence, iron levels in the body must be very tightly regulated.

In an adult male in developed countries, the blood iron level is 4-5g of which 50–60% is associated with hemoglobin. Most iron is stored in the liver, spleen and other organs in ferric (Fe^3+^) form bound to the storage protein, ferritin (FN) [2, 3]. Circulating in plasma is a small amount (3–4 mg) of Fe^3+^ bound to the protein, apo- (*Tf*). The liver senses serum iron through the transferrin receptor 2 pathways and regulates the synthesis and secretion of hepcidin into blood [4, 5]. In body fluids, ferrous (Fe^2+^) ions are highly unstable at physiological pH. Since Fe^2+^ ions are highly reactive, they must be tightly controlled to prevent damage. Therefore, Fe^2+^ is converted to Fe^3+^, which binds to iron chelators (*Tf*, FN) except at very low pH, e.g., inside endosomes [3].

About 25mg of iron is recycled per day via the hemoglobin synthesis and degradation cycle. Only 4–5% of this iron is lost and needs to be replenished through absorption from diet [6, 7]. A schematic representation of the recycling process in iron metabolism has been depicted in literature [8]. Macrophages of the reticulo-endothelial system (RES) degrade senescent red blood cells (RBCs) to release iron that is transported by *Tf*. The maturing erythroblasts in the bone marrow then utilize this iron for incorporation into hemoglobin before entering blood.

Over the last decade, many new details about iron transport have been discovered. Ceruloplasmin (Cp), a ferroxidase associated with macrophage iron release [9–12], is essential in intestinal iron transport. Hepcidin (Hepc), a defensin [13–16], regulates iron release from macrophages through direct degradation of ferroportin (FPN) [17]. There is a complex network of different enzymes and hormones that provides an intricate control of iron metabolism. Teasing out the specific importance of different molecules under different conditions is very challenging. Mathematical modeling of key experimental data can help provide answers to such complex problems.

Mathematical models of iron transport have been developed and refined since the early 1970’s. Most of these models are based on ferrokinetic studies [18, 19] and focus on flux changes and relative abundance of iron in different organs. Also, mathematical models of specific process such as the molecular control of Hepc synthesis or its effect on serum iron have been developed [20, 21]. Typically, models of whole-body iron metabolism consider iron in a single molecular form, which is passively transported according to its concentration gradient. However, iron release from macrophages requires facilitated transport [22] rather than simple passive diffusion [23, 24]. Despite the key role of iron in erythropoiesis [25, 26], models have not considered it in formation of iron hemoglobin. Quantitative understanding of iron metabolism and recycling under normal and different pathological conditions requires mathematical models that integrate mechanistic details of iron uptake, release and transport between the major pools, the dynamics of these processes, as well as the feedback regulation mechanisms controlling them. With a multi-scale model, processes that occur at the molecular and cellular levels can be related to observed behaviors at the tissue-, organ- and whole-body levels.

Recent studies [27–29] propose mathematical models to better predict the dosing strategy of recombinant erythropoietin (rEpo) used to treat anemia in patients with chronic kidney disease (CKD) [30–32]. The current clinical guidelines for treatment of anemia in CKD [33, 34] also include administration of iron dextran to CKD patients where rEpo alone is not enough to improve the hemoglobin levels. Even with low transferrin saturation and low serum iron in patients with CKD anemia, iron uptake through the gut does not increase and oral iron supplements are typically ineffective [7, 33, 35]. The optimal hemoglobin level needs to be personalized [33]. However, current focus on control of overall hemoglobin levels does not account for the impact of therapy on iron metabolism, especially on the recycling process and detrimental effects of iron overload in different tissues [36, 37].

In this study, we have developed a multi-scale model of iron metabolism, which integrates intracellular, molecular mechanisms with cellular and tissue transport of iron. A variety of perturbation scenarios were carefully chosen to estimate model parameters from different parts of the model. Consequently, we can simulate the important clinical outputs (e.g. serum iron, total iron binding capacity, serum hemoglobin, RBC cell concentration) related to therapy while simultaneously providing output of changes in transport fluxes and intracellular species related to iron metabolism. Of special clinical significance is our model simulation of anemia in CKD patients with insufficient erythropoietin and treatments with rEpo and iron dextran infusion. More generally, the goal of this mechanistic mathematical model is to investigate quantitatively the responses of the iron metabolism system under different disease conditions and treatment strategies as a guide for newer and improved treatments.

### Models

**Fig 1:**
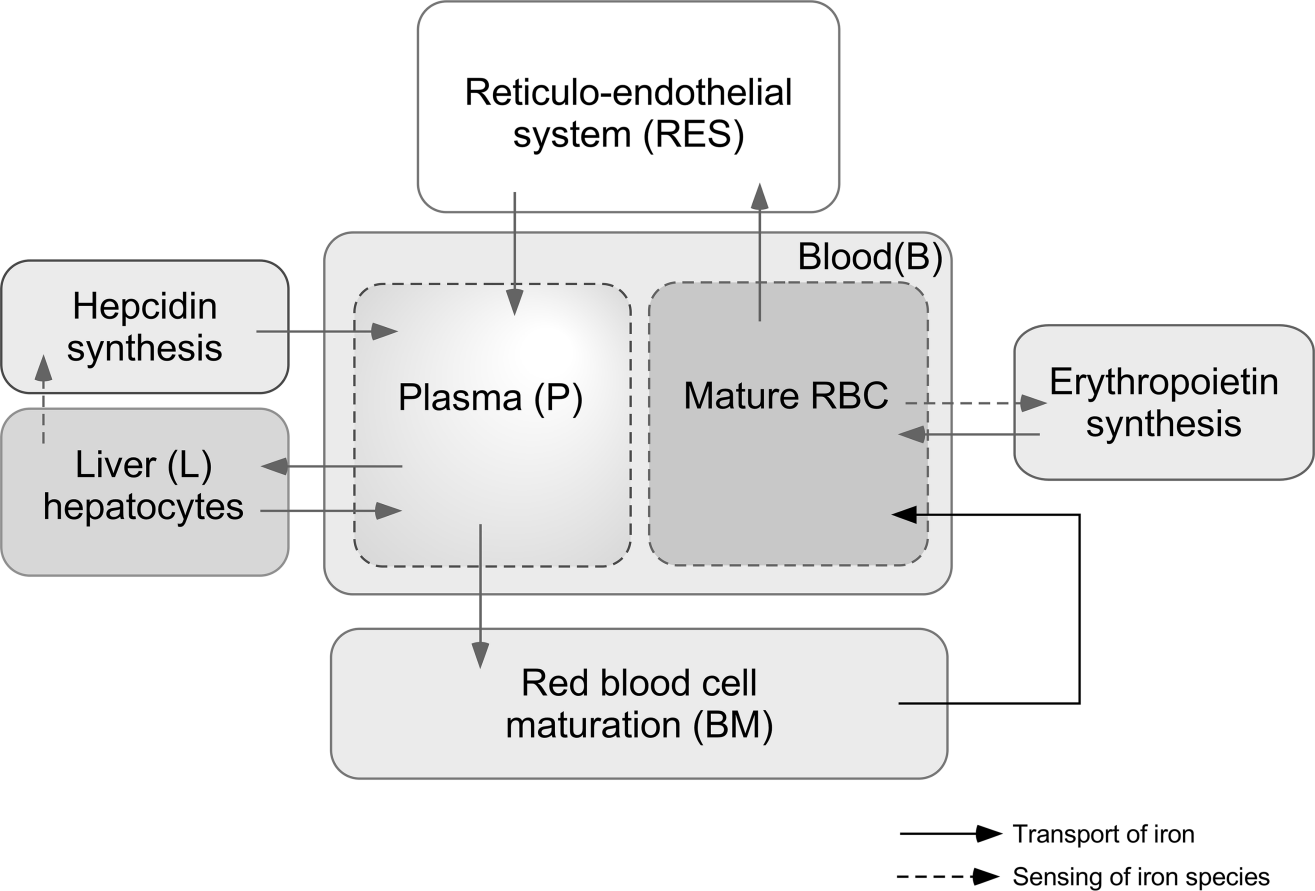
Major components and processes of whole-body iron metabolism incorporated in the model.

The model of iron metabolism developed here has four scales: whole-body, tissue, cellular and molecular. A top-down modeling methodology has been used to develop this model providing just enough detail to simulate the inter-tissue iron fluxes and changes during disease and treatment. A system diagram (Fig 1) shows four major tissue compartments: blood (B), reticular-endothelial system (RES), bone marrow (BM) and liver (L). We have considered erythropoietin (Epo) synthesized by the kidneys and Hepc synthesized from liver as major hormones that help maintain iron homeostasis. The B compartment consists of red blood cell (RBC) and plasma (P) phases. The model covers the major transport processes for iron between these 4 compartments as well as the sensing elements in the liver and kidney.

**Fig 2:**
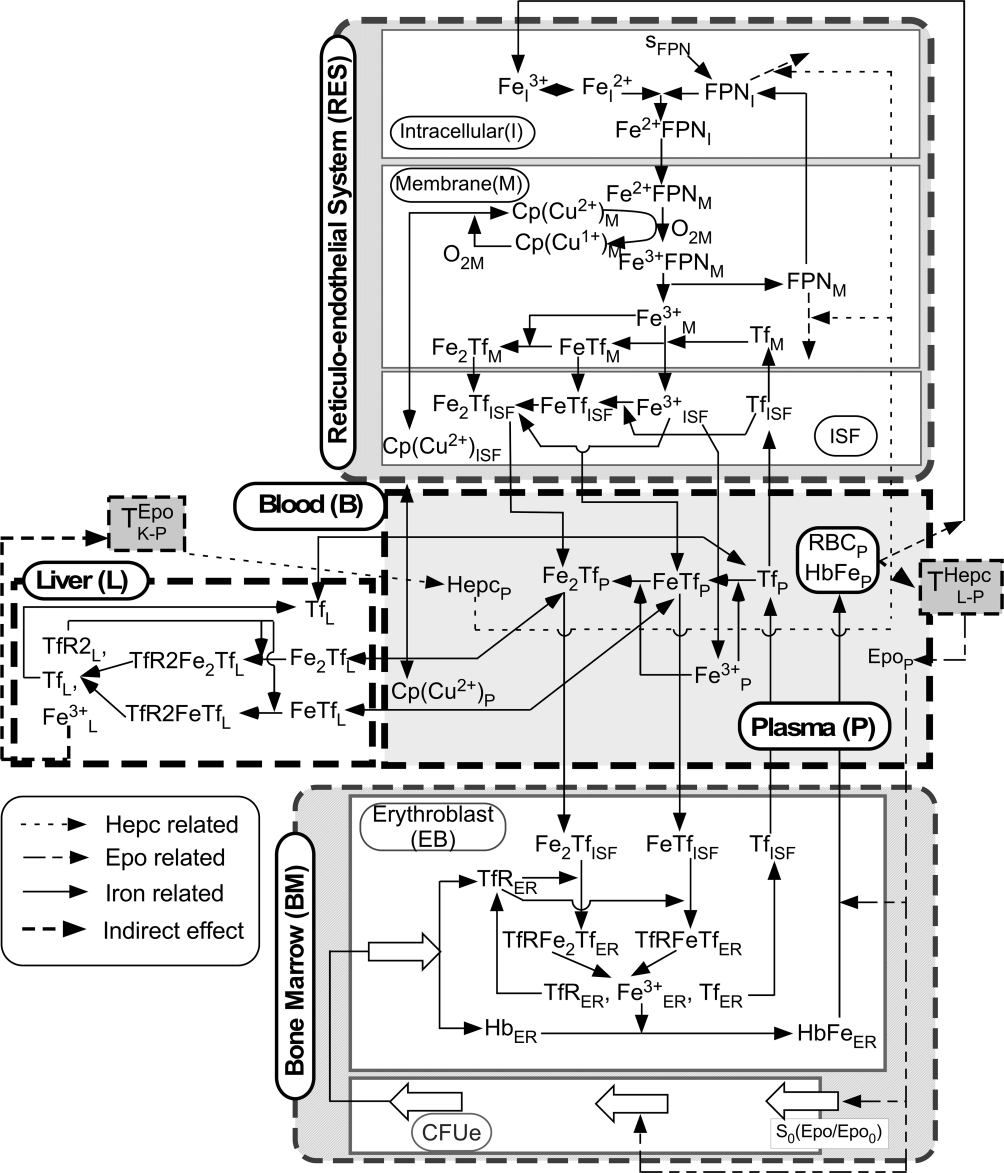
Key transport and reaction processes of iron metabolism involving four model compartments: Blood (B), reticuloendothelial system (RES), bone marrow (BM) and liver (L). Four different types of arrows are used to connect compartments and species which are explained in the legend drawn inside the figure.

A detailed schematic of the reactions and transport processes incorporated in the model is presented in Fig 2. In the RES, we distinguish intracellular (I), membrane (M), and interstitial fluid (ISF) phases. The BM compartment includes colony-forming precursor cells (CFUe) that mature and lead to erythroblasts (EB). The L compartment interfaces with the B compartment. Because the rate of iron loss and replenishment by intestinal absorption is small, this metabolic model focuses on key aspects of iron recycling in contrast to previous models of iron metabolism [18, 20, 26, 28, 38, 39]. Our model incorporates detailed molecular mechanisms of iron transport [18] that differentiate active and passive diffusive transport, as well as the major species of the iron relevant for transport and recycling. These mechanistic details help elucidate the role of different proteins, enzymes and hormones in iron homeostasis and disease conditions.

### Blood (B) compartment

In the blood compartment, we consider RBC dynamics as well as iron-related molecular species in RBC and plasma. In plasma, these species include iron-hemoglobin (*HbFe*), free ferric iron (*Fe*^3+^), apo-transferrin (*Tf*), mono-ferric transferrin (*Fe*^3+^*Tf*), diferric transferrin ((*Fe*^3+^)_2_*Tf*), ceruloplasmin (*Cp*), erythropoietin (*Epo*), and hepcidin (*Hepc*).

In a constant-volume plasma *V*_*P*_, a mass balance of molecular species *j* that diffuses between compartments and changes by reaction rate $R_{P}^{j}$ leads to the plasma concentration $C_{P}^{j}$ dynamics:

$$V_{P}\frac{dC_{P}^{j}}{dt}=J_{ISF\to P}^{j}-J_{P\to EB}^{j}+V_{P}R_{P}^{j}$$ (1)

Here, the ISF- plasma diffusion flux is

$$J_{ISF\to P}^{j}=h_{ISF\to P}^{j}\left(C_{ISF}^{j}-C_{P}^{j}\right)\;j=Tf,(Fe^{3+})Tf,{\left(Fe^{3+}\right)}_{2}Tf,Cp,Fe^{3+}$$ (2)

and the plasma-erythroblast (EB) diffusion flux is

$$J_{P\to EB}^{j}=h_{P\to EB}^{j}\left(C_{P}^{j}-C_{EB}^{j}\right)\;j=Tf,(Fe^{3+})Tf,{\left(Fe^{3+}\right)}_{2}Tf$$ (3)

Where $h_{ISF-P}^{j}$ and $h_{P-EB}^{j}$ are mass transport coefficients; $C_{ISF}^{j}$ and $C_{EB}^{j}$ are concentrations in ISF and EB. Expressions for $R_{P}^{j}$ ($j=Fe^{3+},Tf,\left(Fe^{3+}\right)Tf,{\left(Fe^{3+}\right)}_{2}Tf$ are provided in the Table 1. The degradation of Fe^3+^ refers to non-specific binding of serum Fe^3+^ to plasma.

**Table 1:** Reactions in blood (B) compartment.

Reaction Term	Expression
$R_{P}^{Tf}$	$-k_{Fe^{3+},Tf}C_{P}^{Fe^{3+}}C_{P}^{Tf}$
$R_{P}^{Fe^{3+}Tf}$	$k_{Fe^{3+},Tf}C_{P}^{Fe^{3+}}C_{P}^{Tf}-k_{Fe^{3+},Fe^{3+}Tf}C_{P}^{Fe^{3+}}C_{P}^{Fe^{3+}Tf}$
$R_{P}^{{\left(Fe^{3+}\right)}_{2}Tf}$	$k_{Fe^{3+},Fe^{3+}Tf}C_{P}^{Fe^{3+}}C_{P}^{Fe^{3+}Tf}$
$R_{P}^{Fe^{3+}}$	$-d_{P}^{Fe^{3+}}C_{P}^{Fe^{3+}}$

The concentrations of *Epo* and *Hepc* increase in plasma by endogenous synthesis and decrease by natural degradation:

$$\frac{dC_{P}^{Epo}}{dt}=T_{K\to P}^{Epo}-\frac{C_{P}^{Epo}}{\tau_{}^{Epo}},\;\frac{dC_{P}^{Hepc}}{dt}=T_{L\to P}^{Hepc}-\frac{C_{P}^{Hepc}}{\tau_{}^{Hepc}}$$ (4)

where $T_{K\to P}^{Epo}$ and $T_{L\to P}^{Hepc}$ are the input rates from synthesis of *Epo* by kidneys and *Hepc* by liver, which have been discussed in detail later (section on Erythropoietin and Hepcidin Inputs). These molecular species have average decay times *τ*^*Epo*^ and *τ*^*Hepc*^, respectively.

The RBC number per plasma volume (N_RBC_) increases from EB differentiation and decreases by macrophage phagocytosis represented by a cell number density balance:

$$\frac{dN_{RBC}}{dt}=\frac{k_{RBC\leftarrow EB}}{V_{P}}N_{EB}-d_{RES\leftarrow RBC}N_{RBC}$$ (5)

where *N*_*EB*_ is the EB number in the bone marrow compartment, *k*_*RBC←EB*_ is the differentiation rate coefficient. The death rate coefficient by RES phagocytosis *d*_*RES*←*RBC*_ = 1/*τ*_*RBC*_ is the inverse of the average RBC life-span in plasma.

Associated with the RBC is *HbFe*^3+^, whose concentration (relative to plasma volume) changes by the entry of differentiating erythroblasts carrying *HbFe*^3+^ and decreases with the removal of RBC by phagocytosis:

$$\frac{dC_{P}^{HbFe}}{dt}=\frac{k_{RBC\leftarrow EB}}{V_{P}}\left(\frac{N_{EB}}{N_{EB}^{SS}}\right)C_{EB}^{HbFe}-d_{RES\leftarrow RBC}C_{P}^{HbFe}$$ (6)

where $C_{EB}^{HbFe}$ is the *HbFe*^3+^ concentration in bone marrow. Here, the number of EB cells in the bone marrow is scaled by their steady-state values, which are initial values, $N_{EB}^{SS}=N_{EB}\left(0\right)$.

### Bone Marrow (BM) compartment

#### Precursor cell (CFUe) dynamics

The CFUe number *N*_*CFU*_(*μ*,*t*) changes with respect to maturity, i.e., age (*μ*) and time (*t*) [38]. These cells proliferate, but are negligible in bone marrow reaction or transport processes related to iron. From an age-distributed cell number balance, we obtain

$$\frac{\partial N_{CFU}}{\partial t}+v_{CFU}\frac{\partial N_{CFU}}{\partial \mu }=\beta_{CFU}N_{CFU};\;0\leq \mu \leq \mu_{F}$$ (7)

where the rate of aging is *ν*_*CFU*_ = *dμ*/*dt* and the rate coefficient of proliferation *β*_*CFU*_ is assumed constant. This is a simplification of a model presented by Mahaffy et al. [25]. At steady state, CFUe number is

$$N_{CFU}^{SS}(\mu )=N_{CFU}^{0}\exp(\beta_{CFU}\mu )$$ (8)

The rate of CFUe aging is represented as:

$$v_{CFU}=v_{0}\left(\frac{C_{P}^{Epo}}{C_{P}^{Epo}+Km_{Epo}}\right)$$ (9)

This relation is based on saturation of high affinity Epo receptors of CFUe.

The CFUe number forming at *μ* = 0 from differentiating Burst Forming Unit Erythroids (BFUe) is dependent on the total BFUe number *N*_*BFUe*_ available for differentiation and $C_{P}^{Epo}(t)$ according to an empirical function [36]:

$$N_{CFU}\left(0,t\right)=\{\begin{array}{ccc} \begin{array}{c} N_{BFUe}\left(\frac{C_{P}^{Epo}\left(t\right)}{C_{P}^{Epo}\left(0\right)}\right)\\ N_{BFUe} \end{array} & \begin{array}{c} for\\ for \end{array} & \begin{array}{c} C_{P}^{Epo}(t)<C_{P}^{Epo}(0)\\ C_{P}^{Epo}(t)\geq C_{P}^{Epo}(0) \end{array} \end{array}$$ (10)

where $C_{P}^{Epo}(0)$ is the steady-state value.

No iron transport happens in the CFUe age-distributed compartment. Furthermore, all details of the development of the receptors on the CFUe etc. are ignored for this model. At maturity CFUe’s become erythroblasts (EB) with the average number of transferrin receptors on their surface and the average intracellular iron-free hemoglobin. The internal processes are developed for recycling of transferrin receptors and incorporation of iron into hemoglobin.

#### Erythroblast (EB) region relations

At full maturity *μ*_*F*_, differentiation to EB occurs at a rate:

$$F_{EB\leftarrow CFU}=v_{CFU}{\frac{\partial N_{CFU}}{\partial \mu }|}_{\mu =\mu_{F}}$$ (11)

The EB number increases by CFUe differentiation and decreases by EB differentiation into RBC:

$$\frac{dN_{EB}}{dt}=F_{EB\leftarrow CFU}-k_{RBC\leftarrow EB}N_{EB}$$ (12)

Where *k*_*RBC*←*EB*_ is a differentiation rate coefficient. The process of maturation of erythroblasts into mature RBC has been simplified into a lumped model unlike the age-distributed model for maturation of CFU. With this simplification, iron uptake by maturing erythroblasts is represented by a single set of reactions in a single EB compartment. At steady state, we obtain

$$N_{EB}^{SS}=\frac{V_{P}d_{RES\leftarrow RBC}}{k_{RBC\leftarrow EB}}N_{RBC}^{SS}$$ (13)

Combining equations above, we find

$$F_{EB\leftarrow CFU}^{SS}=\nu_{CFU}{\frac{\partial N_{CFU}}{\partial \mu }|}_{\mu =\mu_{F}}=\nu_{CFU}\beta_{CFU}N_{CFU}^{SS}\left(\mu_{F}\right)=k_{RBC\leftarrow EB}N_{EB}^{SS}$$ (14)

Defining the characteristic time for CFUe’s to become RBC’s as *τ*_*EB*_ = 1/*k*_*RBC*←*EB*_, we can express the steady-state CFU number as follows:

$$N_{CFU}^{SS}\left(\mu_{F}\right)=N_{EB}^{SS}/\left(\tau_{RBC}\nu_{CFU}\beta_{CFU}\right)$$ (15)

The steady-state CFU number is defined as follows:

$$N_{EB}^{SS}=\frac{V_{P}d_{RES\leftarrow RBC}}{k_{RBC\leftarrow EB}}N_{RBC}^{SS}=V_{P}d_{RES\leftarrow RBC}\tau_{EB}N_{RBC}^{SS}$$ (16)

To compute $N_{CFU}^{SS}\left(\mu_{F}\right)$ and $N_{EB}^{SS}$, we obtain estimates from the literature for *V*_*p*_, *d*_*RES*←*RBC*_, *τ*_*EB*_, *β*_*CFU*_ and $N_{RBC}^{SS}$. Following Mahaffy [25], we set *ν*_*CFU*0_ = 1.

#### Iron and transferrin dynamics

In the EB region, iron is taken from (*Fe*^3+^)*Tf* and (*Fe*^3+^)_2_*Tf* via transferrin receptor (*TfR)*. The reactions of chemical species are represented by the following kinetics (Fig 2):

$$\begin{array}{l} TfR+(Fe^{3+})Tf↔(TfR)Fe^{3+}Tf\\ TfR+{\left(Fe^{3+}\right)}_{2}Tf↔(TfR){\left(Fe^{3+}\right)}_{2}Tf\\ (TfR)(Fe^{3+})Tf\to TfR+Fe^{3+}+Tf\\ (TfR){\left(Fe^{3+}\right)}_{2}Tf\to TfR+2Fe^{3+}+Tf\\ Fe^{3+}+Hb\to HbFe^{3+} \end{array}$$ (17)

Here, the mechanism of iron release from *Tf* and recycling of *Tf* and *TfR* has been simplified as a single reaction.

In representing the concentration changes of species *j* in EB with time, we assume EB volume is constant. Concentrations in EB increases with CFUe entry and decreases because of EB maturation into RBC and reactions in the EB compartment as follows:

$$\frac{dC_{EB}^{j}}{dt}=\frac{F_{EB\leftarrow CFU}B_{CFU}^{j}}{V_{EB}}-\frac{k_{RBC\leftarrow EB}}{V_{EB}}\left(\frac{N_{EB}}{N_{EB}^{SS}}\right)C_{EB}^{j}+R_{EB}^{j},\;j=TfR,Hb$$ (18)

where $B_{CFU}^{TfR}$, $B_{CFU}^{Hb}$ are the constant amounts of *TfR* and *Hb* per CFUe entering the EB compartment [7, 40].

Since *HbFe*^3+^ is not in CFUe its concentration changes according to

$$\frac{dC_{EB}^{HbFe^{3+}}}{dt}=\frac{-k_{RBC\leftarrow EB}}{V_{EB}}\left(\frac{N_{EB}}{N_{EB}^{SS}}\right)C_{EB}^{HbFe^{3+}}+R_{EB}^{HbFe^{3+}}$$ (19)

For the species that do not enter or leave the EB compartment, the concentration changes only by reaction:

$$\frac{dC_{EB}^{j}}{dt}=R_{EB}^{j},\;j=(TfR)(Fe^{3+})Tf,(TfR){\left(Fe^{3+}\right)}_{2}Tf,Fe^{3+}$$ (20)

For other species concentrations, changes can occur by reaction and transport into or out of plasma:

$$\frac{dC_{EB}^{j}}{dt}=R_{EB}^{j}+\frac{J_{EB\leftarrow P}}{V_{EB}},\;j=(Fe^{3+})Tf,{\left(Fe^{3+}\right)}_{2}Tf,Tf$$ (21)

Expressions for $R_{EB}^{j}$ are provided in the Table 2.

**Table 2:** Reactions in the erythroblast (EB) compartment.

Reaction Term	Expression
$R_{EB}^{TfR}$	$\begin{array}{l} -k_{on,TfR,Fe^{3+}Tf}C_{EB}^{TfR}C_{Eb}^{Fe^{3+}Tf}-k_{on,TfR,{\left(Fe^{3+}\right)}_{2}Tf}C_{EB}^{TfR}C_{EB}^{{\left(Fe^{3+}\right)}_{2}Tf}+k_{TfR,recycle}\left(C_{EB}^{TfRFe^{3+}Tf}+C_{EB}^{TfR{\left(Fe^{3+}\right)}_{2}Tf}\right)\\ +k_{off,TfRFe^{3+}Tf}C_{EB}^{TfRFe^{3+}Tf}+k_{off,TfR{\left(Fe^{3+}\right)}_{2}Tf}C_{EB}^{TfR{\left(Fe^{3+}\right)}_{2}Tf} \end{array}$
$R_{EB}^{TfRFe^{3+}Tf}$	$k_{on,TfR,Fe^{3+}Tf}C_{EB}^{TfR}C_{EB}^{Fe^{3+}Tf}-k_{off,TfRFe^{3+}Tf}C_{EB}^{TfRFe^{3+}Tf}-k_{TfR,recycle}C_{EB}^{TfRFe^{3+}Tf}$
$R_{EB}^{TfR{\left(Fe^{3+}\right)}_{2}Tf}$	$k_{on,TfR,{\left(Fe^{3+}\right)}_{2}Tf}C_{EB}^{TfR}C_{EB}^{{\left(Fe^{3+}\right)}_{2}Tf}-k_{off,TfR{\left(Fe^{3+}\right)}_{2}Tf}C_{EB}^{TfR{\left(Fe^{3+}\right)}_{2}Tf}-k_{TfR,recycle}C_{EB}^{TfR{\left(Fe^{3+}\right)}_{2}Tf}$
$R_{EB}^{Fe^{3+}Tf}$	$-k_{on,TfR,Fe^{3+}Tf}C_{EB}^{TfR}C_{EB}^{Fe^{3+}Tf}+k_{off,TfRFe^{3+}Tf}C_{EB}^{TfRFe^{3+}Tf}$
$R_{EB}^{{\left(Fe^{3+}\right)}_{2}Tf}$	$-k_{on,TfR,{\left(Fe^{3+}\right)}_{2}Tf}C_{EB}^{TfR}C_{EB}^{{\left(Fe^{3+}\right)}_{2}Tf}+k_{off,TfR{\left(Fe^{3+}\right)}_{2}Tf}C_{EB}^{TfR{\left(Fe^{3+}\right)}_{2}Tf}$
$R_{EB}^{Fe^{3+}}$	$k_{TfR,recycle}\left(C_{EB}^{TfRFe^{3+}Tf}+C_{EB}^{TfR{\left(Fe^{3+}\right)}_{2}Tf}\right)-k_{Fe^{3+},Hb}C_{EB}^{Fe^{3+}}C_{EB}^{Hb}$
$R_{EB}^{Hb}$	$-k_{Fe^{3+},Hb}C_{EB}^{Fe^{3+}}C_{EB}^{Hb}$
$R_{EB}^{HbFe^{3+}}$	$k_{Fe^{3+},Hb}C_{EB}^{Fe^{3+}}C_{EB}^{Hb}$

### RES compartment

The RES compartment is divided into intracellular (I), membrane (M) and interstitial (ISF) regions, which are assumed to have constant volumes (Fig 2). Iron from the hemoglobin in senescent RBC’s that are phagocytosed by the macrophages of the ISF is delivered as *Fe*^3+^ into the intracellular region. Then, *Fe*^3+^ is converted to *Fe*^2+^ (labile iron pool) by endosomal reducing agents and then it binds to intracellular FPN. This complex is carried by an energy-driven process to the cell membrane, where *Fe*^2+^ is converted to *Fe*^3+^ by sequestration with ferritin (FN). At the membrane, oxygen (*O*_2_) and ceruloplasmin (*CpCu*^2+^) oxidize *Fe*^2+^ *FPN* to *Fe*^3+^ *FPN*. The dissociation of *Fe*^3+^ *FPN* allows FPN to recycle and *Fe*^3+^ to diffuse into the ISF or bind to *Tf* to form *Fe*^3+^*Tf* in the membrane region.

For most species, the transport fluxes between M and ISF are governed by passive diffusion:

$$J_{M\to ISF}^{j}=h_{M-ISF}^{j}\left(C_{M}^{j}-C_{ISF}^{j}\right),\;j=Tf,(Fe^{3+})Tf,{\left(Fe^{3+}\right)}_{2}Tf$$ (22)

For a few species, the transport fluxes between RES regions are energy-driven processes:

$$J^{FPN}{}_{I\leftarrow M}=h^{}{}_{I\leftarrow M}C_{M}^{FPN},\;J^{Fe^{2+}FPN}{}_{M\leftarrow I}=h^{}{}_{M\leftarrow I}C_{I}^{Fe^{2+}FPN},\;J^{Fe^{3+}}{}_{ISF\leftarrow M}=h^{}{}_{ISF\leftarrow M}C_{M}^{Fe^{3+}}$$ (23)

In the I region, the species *j* concentrations change according to

$$\frac{dC_{I}^{j}}{dt}=\frac{J^{j}{}_{I\leftarrow M}-J^{j}{}_{M\leftarrow I}}{V_{I}}+R_{I}^{j}\;j=Fe^{2+},FPN$$ (24)

The *Fe*^3+^ from the senescent RBC acts as a source of *Fe*^3+^ in the intracellular (I) region. Hence the equation for concentration of *Fe*^3+^ in the I region varies as:

$$\frac{dC_{I}^{Fe^{3+}}}{dt}=\frac{J^{Fe^{3+}}{}_{I\leftarrow M}-J^{Fe^{3+}}{}_{M\leftarrow I}}{V_{I}}+R^{Fe^{3+}}{}_{I}+d_{RES\leftarrow RBC}C_{P}^{HbFe}\left(\frac{V_{P}}{V_{I}}\right)$$ (25)

The FPN concentration depends on FPN endogenous synthesis and loss:

$$\frac{dC_{I}^{FPN}}{dt}=\frac{J^{FPN}{}_{I\leftarrow M}}{V_{I}}+R_{I}^{FPN}+S_{I}^{FPN}$$ (26)

where

$$S_{I}^{FPN}=\frac{C_{I}^{FPN}{|}_{SS}-C_{I}^{FPN}}{\tau_{FPN}}$$ (27)

Here, *τ*_*FPN*_ is the half-life of FPN and $C_{I}^{FPN}{|}_{SS}$ is the steady-state concentration of FPN. The model ignores any transcriptional regulation of FPN as has been observed especially due to hypoxia, iron deficiency etc. [41, 42]. In the M region, the species *j* concentrations change as:

$$\frac{dC_{M}^{j}}{dt}=\frac{J_{M\leftarrow I}^{j}+J_{M\leftarrow ISF}^{j}-J_{I\leftarrow M}^{j}}{V_{M}}+R^{j}{}_{M}$$ (28)

where $j=Fe^{2+}FPN,Fe^{3+}FPN,Fe^{3+},FPN,Cp^{Cu^{2+}},Cp^{Cu^{1+}},Tf,Fe^{3+}Tf,Fe_{2}^{3+}Tf$. In the ISF region, the species *j* concentration changes as:

$$\frac{dC_{ISF}^{j}}{dt}=\frac{N_{scale\_factor}J_{ISF\leftarrow M}^{j}-J_{ISF\to P}^{j}}{V_{ISF}}+R^{j}{}_{ISF}$$ (29)

$$\mathrm{where}\;J_{ISF\to P}^{j}=h_{ISF-P}^{j}\left(C_{ISF}^{j}-C_{P}^{j}\right),\;j=Tf,\;(Fe^{3+})Tf,\;{\left(Fe^{3+}\right)}_{2}Tf,Cp^{Cu^{2+}},Cp^{Cu^{1+}}$$ (30)

The reaction rates for each chemical species *j* in the three RES regions (*R*^*j*^_*ISF*_,*R*^*j*^_*I*_,*R*^*j*^_*M*_) are based on the kinetics as indicated in Fig 2 and described below. We incorporated the *Hepc* blocking of iron transport from RES through degradation of both intracellular and membrane FPN [15–17]. In the intracellular (I) region:

$$\begin{array}{l} Fe^{3+}↔Fe^{2+}\\ Fe^{2+}+FPN\to Fe^{2+}FPN\\ FPN+Hepc\to ⊗\\ FPN\to ⊗ \end{array}$$ (31)

where ⊗ represents degradation products. In the membrane (M) region:

$$\begin{array}{l} 4(Fe^{2+})FPN+O_{2}+4H^{+}\overset{}{\underset{}{⇄}}4(Fe^{3+})FPN+2H_{2}O\\ Fe^{2+}FPN+Cp\left(Cu^{2+}\right)\to Fe^{3+}FPN+Cp\left(Cu^{1+}\right)\\ Fe^{3+}FPN\to Fe^{3+}+FPN\\ Fe^{3+}+Tf\to (Fe^{3+})Tf\\ Fe^{3+}+(Fe^{3+})Tf\to {\left(Fe^{3+}\right)}_{2}Tf\\ 4Cp(Cu^{1+})+O_{2}+4H^{+}\overset{}{\to }4Cp(Cu^{2+})+2H_{2}O\\ FPN+Hepc\to ⊗ \end{array}$$ (32)

In the interstitial (ISF) region:

$$\begin{array}{l} Fe^{3+}+Tf\to (Fe^{3+})Tf\\ Fe^{3+}+Fe^{3+}Tf\to {\left(Fe^{3+}\right)}_{2}Tf\\ 4Cp(Cu^{1+})+O_{2}+4H^{+}\overset{}{\to }4Cp(Cu^{2+})+2H_{2}O \end{array}$$ (33)

Expressions for $R_{I}^{j},R_{M}^{j},R_{ISF}^{j}$ are provided in Table 3.

**Table 3:** Reactions in the RES (I, M, ISF) compartment.

Reaction Terms	Expressions
$R_{I}^{Fe^{2+}}$	$-k_{Fe^{2+}\to Fe^{3+}}C_{I}^{Fe^{2+}}+k_{Fe^{3+}\to Fe^{3+}}C_{I}^{Fe^{3+}}-k_{Fe^{2+},FPN}C_{I}^{Fe^{2+}}C_{I}^{FPN}$
$R_{I}^{Fe^{3+}}$	$k_{Fe^{2+}\to Fe^{3+}}C_{I}^{Fe^{2+}}-k_{Fe^{3+}\to Fe^{3+}}C_{I}^{Fe^{3+}}$
$R_{I}^{Fe^{2+}FPN}$	$k_{Fe^{2+},FPN}C_{I}^{Fe^{2+}}C_{I}^{FPN}$
$R_{I}^{FPN}$	$-k_{Fe^{2+},FPN}C_{I}^{Fe^{2+}}C_{I}^{FPN}-k_{FPN,Hepc}C_{I}^{FPN}C_{P}^{Hepc}-k_{FPN,rhHepc}C_{I}^{FPN}C_{T}^{rhHepc}$
$R_{M}^{Fe^{2+}FPN}$	$-\left(k_{Fe^{2+}FPN,O_{2}}C_{M}^{O_{2}}+k_{Fe^{2+}FPN,Cp}C_{M}^{Cp(Cu^{2+})}\right)C_{M}^{Fe^{2+}FPN}$
$R_{M}^{Fe^{3+}FPN}$	$\left(k_{Fe^{2+}FPN,O_{2}}C_{M}^{O_{2}}+k_{Fe^{2+}FPN,Cp}C_{M}^{Cp(Cu^{2+})}\right)C_{M}^{Fe^{2+}FPN}-k_{Fe^{3+}FPN,Fe^{3+}}C_{M}^{Fe^{3+}FPN}$
$R_{M}^{Fe^{3+}}$	$k_{Fe^{3+}FPN,Fe^{3+}}C_{M}^{Fe^{3+}FPN}-k_{Fe^{3+},Tf}C_{M}^{Fe^{3+}}C_{M}^{Tf}-k_{Fe^{3+},Fe^{3+}Tf}C_{M}^{Fe^{3+}}C_{M}^{Fe^{3+}Tf}$
$R_{M}^{FPN}$	$k_{Fe^{3+}FPN,Fe^{3+}}C_{M}^{Fe^{3+}FPN}-k_{FPN,Hepc}C_{M}^{FPN}C_{M}^{Hepc}-k_{FPN,rhHepc}C_{M}^{FPN}C_{M}^{rhHepc}$
$R_{M}^{Fe^{3+}Tf}$	$k_{Fe^{3+},Tf}C_{M}^{Fe^{3+}}C_{M}^{Tf}-k_{Fe^{3+},Fe^{3+}Tf}C_{M}^{Fe^{3+}}C_{M}^{Fe^{3+}Tf}$
$R_{M}^{{\left(Fe^{3+}\right)}_{2}Tf}$	$k_{Fe^{3+},Fe^{3+}Tf}C_{M}^{Fe^{3+}}C_{M}^{Fe^{3+}Tf}$
$R_{M}^{Tf}$	$-k_{Fe^{3+},Tf}C_{M}^{Fe^{3+}}C_{M}^{Tf}$
$R_{M}^{Cp\left(Cu^{2+}\right)}$	$-k_{Fe^{2+}FPN,Cp}C_{M}^{Cp(Cu^{2+})}C_{M}^{Fe^{2+}FPN}+k_{Cp\left(Cu^{1+}\right),O_{2}}C_{M}^{O_{2}}C_{M}^{Cp(Cu^{1+})}$
$R_{M}^{Cp\left(Cu^{1+}\right)}$	$k_{Fe^{2+}FPN,Cp}C_{M}^{Cp(Cu^{2+})}C_{M}^{Fe^{2+}FPN}-k_{Cp\left(Cu^{1+}\right),O_{2}}C_{M}^{O_{2}}C_{M}^{Cp(Cu^{1+})}$
$R_{ISF}^{Fe^{3+}Tf}$	$k_{Fe^{3+},Tf}C_{ISF}^{Fe^{3+}}C_{ISF}^{Tf}-k_{Fe^{3+},Fe^{3+}Tf}C_{ISF}^{Fe^{3+}}C_{ISF}^{Fe^{3+}Tf}$
$R_{ISF}^{{\left(Fe^{3+}\right)}_{2}Tf}$	$k_{Fe^{3+},Fe^{3+}Tf}C_{ISF}^{Fe^{3+}}C_{ISF}^{Fe^{3+}Tf}$
$R_{ISF}^{Tf}$	$-k_{Fe^{3+},Tf}C_{ISF}^{Fe^{3+}}C_{ISF}^{Tf}$

### Liver (L) compartment

In the liver compartment, we consider the dynamics of binding of iron-transferrin to transferrin receptor 2 (*TfR2*), internalization and storage of iron inside the liver depicted in Fig 2. This compartment deals with the changes in serum iron and its effect on the extent of iron stored (Fe^3+^) in the liver, and subsequently on hepcidin synthesis and secretion. The other functions of the liver in iron metabolism, including storage and release of iron from ferritin, are lumped into the RES compartment in this model rather than the L compartment. The very low binding affinity of transferrin to *TfR2* produces significantly different dynamics in this compartment as opposed to assuming the same binding affinity as *TfR*.

All species transport between plasma and liver is governed by passive diffusion:

$$J_{P\to L}^{j}=h_{P\to L}^{j}\left(C_{P}^{j}-C_{L}^{j}\right)j=Tf,(Fe^{3+})Tf,{\left(Fe^{3+}\right)}_{2}Tf$$ (34)

Inside the liver compartment of volume *V*_*L*_, the concentrations of some species change as:

$$\frac{dC_{L}^{j}}{dt}=\frac{J_{P\to L}^{j}}{V_{L}}+R_{L}^{j},\;j=Tf,(Fe^{3+})Tf,{\left(Fe^{3+}\right)}_{2}Tf$$ (35)

whereas others change as:

$$\frac{dC_{L}^{j}}{dt}=R_{L}^{j},\;j=TfR2,TfR2(Fe^{3+})Tf,TfR2{\left(Fe^{3+}\right)}_{2}Tf,Fe^{3+}$$ (36)

Based on the following chemical reactions, equations for reaction rates $R_{L}^{j}$ are provided in Table 4.

**Table 4:** Reaction terms in liver (L) compartment.

Reaction Term	Expression
$R_{L}^{TfR2}$	$-k_{on,TfR2,Fe^{3+}Tf}C_{L}^{TfR2}C_{L}^{Fe^{3+}Tf}-k_{on,TfR2,{\left(Fe^{3+}\right)}_{2}Tf}C_{L}^{TfR2}C_{L}^{{\left(Fe^{3+}\right)}_{2}Tf}+k_{TfR,recycle}\left(C_{L}^{TfR2Fe^{3+}Tf}+C_{L}^{TfR2{\left(Fe^{3+}\right)}_{2}Tf}\right)$ $+k_{off,TfR2Fe^{3+}Tf}C_{L}^{TfR2Fe^{3+}Tf}+k_{off,TfR2{\left(Fe^{3+}\right)}_{2}Tf}C_{L}^{TfR2{\left(Fe^{3+}\right)}_{2}Tf}$
$R_{L}^{TfR2Fe^{3+}Tf}$	$k_{on,TfR2,Fe^{3+}Tf}C_{L}^{TfR2}C_{L}^{Fe^{3+}Tf}-k_{off,TfR2Fe^{3+}Tf}C_{L}^{TfR2Fe^{3+}Tf}-k_{TfR2,recycle}C_{L}^{TfR2Fe^{3+}Tf}$
$R_{L}^{TfR2{\left(Fe^{3+}\right)}_{2}Tf}$	$k_{on,TfR2,{\left(Fe^{3+}\right)}_{2}Tf}C_{L}^{TfR2}C_{L}^{{\left(Fe^{3+}\right)}_{2}Tf}-k_{off,TfR2{\left(Fe^{3+}\right)}_{2}Tf}C_{L}^{TfR2{\left(Fe^{3+}\right)}_{2}Tf}-k_{TfR2,recycle}C_{L}^{TfR2{\left(Fe^{3+}\right)}_{2}Tf}$
$R_{L}^{Fe^{3+}Tf}$	$-k_{on,TfR2,Fe^{3+}Tf}C_{L}^{TfR2}C_{L}^{Fe^{3+}Tf}+k_{off,TfR2Fe^{3+}Tf}C_{L}^{TfR2Fe^{3+}Tf}$
$R_{L}^{{\left(Fe^{3+}\right)}_{2}Tf}$	$-k_{on,TfR2,{\left(Fe^{3+}\right)}_{2}Tf}C_{L}^{TfR2}C_{L}^{{\left(Fe^{3+}\right)}_{2}Tf}+k_{off,TfR2{\left(Fe^{3+}\right)}_{2}Tf}C_{L}^{TfR2{\left(Fe^{3+}\right)}_{2}Tf}$
$R_{L}^{Fe^{3+}}$	$k_{TfR2,recycle}\left(C_{L}^{TfR2Fe^{3+}Tf}+C_{L}^{TfR2{\left(Fe^{3+}\right)}_{2}Tf}\right)-d_{L,Fe^{3+}}C_{EB}^{Fe^{3+}}$

$$\begin{array}{l} TfR2+(Fe^{3+})Tf↔(TfR2)(Fe^{3+})Tf\\ TfR2+{\left(Fe^{3+}\right)}_{2}Tf↔(TfR2){\left(Fe^{3+}\right)}_{2}Tf\\ (TfR2)(Fe^{3+})Tf\to TfR2+Fe^{3+}+Tf\\ (TfR2){\left(Fe^{3+}\right)}_{2}Tf\to TfR2+2Fe^{3+}+Tf\\ Fe^{3+}\to ⊗ \end{array}$$ (37)

where ⊗ represents degradation products. In this case, degradation refers to binding or chelation of Fe^3+^ (possibly into Ferritin or other forms).

The affinity of *TfR2* for (*Fe*^3+^)*Tf* and (*Fe*^3+^)_2_*Tf* is known to be 30 times less than *TfR* which is used to compute the rate of binding of (*Fe*^3+^)*Tf* and (*Fe*^3+^)_2_*Tf* to *TfR*2 from the binding rates to *TfR*

$$\begin{array}{l} k_{on,TfR2,Fe^{3+}Tf}=k_{on,TfR,Fe^{3+}Tf}*\left(k_{D,TfR,Fe^{3+}Tf}/\eta \right)\\ k_{on,TfR2,{\left(Fe^{3+}\right)}_{2}Tf}=k_{on,TfR,{\left(Fe^{3+}\right)}_{2}Tf}*\left(k_{D,TfR,{\left(Fe^{3+}\right)}_{2}Tf}/\eta \right) \end{array}$$ (38)

*η* = 30 is the ratio of the affinity monoferric and diferric transferrin to *TfR* and *TfR*2

### Erythropoietin and hepcidin inputs

In addition to endogenous inputs of erythropoietin and Hepc to this model, these inputs can be exogenous for therapy. Whereas the endogenous input rates are empirical, the exogenous input rates are based on a pharmacokinetic model (S1 Fig). The equations for both endogenous Epo and Hepc inputs are developed in this section, while those for exogenous inputs are described later in “**System perturbations for parameter estimation”**

#### Endogenous erythropoietin entry rate

The rate of erythropoietin (Epo) entry into plasma is directly related to its synthesis rate in the renal cortex [43], which depends on many factors [44]. In this model, it is assumed that control of Epo synthesis is primarily a function of *HbFe*^3+^. Data from previous studies [39, 45, 46] were used to develop an empirical relationship between $C_{P}^{HbFe}$ and Epo synthesis rate, which becomes the Epo entry rate into plasma:

$$T_{K\to P}^{Epo}=\left(C_{0}^{Epo}/\tau^{Epo}\right)\left\{1+\alpha^{Epo}\exp\left[\beta^{Epo}\left(C_{P}^{HbFe}{|}_{SS}-C_{P}^{HbFe}\right)\right]\right\}/(1+\alpha^{Epo})$$ (39)

Here, *α*^*Epo*^, *β*^*Epo*^, $C_{0}^{Epo}$ are model parameters.

#### Endogenous Hepcidin entry rate

The rate of Hepc entry into plasma is directly related to its synthesis rate in liver, which is sensitive to serum iron levels. A complex intracellular network of pathways provides precise control of Hepc [47, 48]. The variant of transferrin receptor, *TfR*2, which is found abundantly on hepatocytes has been shown to be key to regulation of Hepc synthesis along with other surface proteins like HFE [14, 49]. In our model, the transferrin receptor 2 (*TfR*2) pathway mediates uptake of iron from plasma iron transferrin. The dynamics of hepatocyte iron are described in the Liver compartment section. To represent the time delay from a change in serum transferrin saturation or serum iron to the appearance of Hepc in plasma, an intermediate species *IHepc* is incorporated in the model. Changes in concentration of *IHepc* can be rationalized as representing the dynamics of normalized mRNA expression for Hepc. The *IHepc* concentration increases by synthesis at rate *R*^*IHepc*^ and decreases by natural mRNA degradation with a characteristic time *τ*^*IHepc*^:

$$\frac{dC_{}^{IHepc}}{dt}=R_{}^{IHepc}-\frac{C_{}^{IHepc}}{\tau^{IHepc}}$$ (40)

At steady-state, ${C_{}^{IHepc}|}_{SS}=\left({R_{}^{IHepc}|}_{SS}\right)\tau^{IHepc}$

The rate of entry of Hepc into the BL compartment is defined as:

$$T_{L\to P}^{Hepc}=k_{IHepc,Hepc}C^{IHepc}\Rightarrow k_{IHepc,Hepc}=\frac{{T_{L\to P}^{Hepc}|}_{SS}}{{C^{IHepc}|}_{SS}}$$ (41)

At steady-state, the rate of entry to the Hepc concentration is derived from Eq (41) as

$${T_{L\to P}^{Hepc}|}_{SS}=C_{0}^{Hepc}|/\tau_{Hepc}$$

The rate of synthesis of *IHepc* depends on whether intracellular *Fe*^3+^ in the L compartment is less or more than its steady-state level. Increase in liver intracellular *Fe*^3+^ leads to increase in Hepc [50] through increased transcription which is represented in the model as increased synthesis of IHepc [51]. In the model, the transcriptional regulation is represented as a Hill’s function. Lowering of liver *Fe*^3+^ leads to inhibition of IHepc synthesis as a function of the reduction of liver *Fe*^3+^ from the steady-state concentration:

$$R^{IHepc}=\{\begin{array}{c} k_{IHepc,Fe^{3+}}\left({\left(C_{L}^{Fe^{3+}}\right)}^{n_{IHepc,Fe^{3+}}}/\left({\left(C_{L}^{Fe^{3+}}\right)}^{n_{IHepc,Fe^{3+}}}+{\left(km_{IHepc,Fe^{3+}}\right)}^{n_{IHepc,Fe^{3+}}}\right)\right)\\ {R^{IHepc}|}_{SS}/\left(1+kI_{IHepc,Fe^{3+}}\left({C_{L}^{Fe^{3+}}|}_{SS}-C_{L}^{Fe^{3+}}\right)\right) \end{array}\begin{array}{cc} & for\\ & for \end{array}\begin{array}{cc} & C_{L}^{Fe^{3+}}>{C_{L}^{Fe^{3+}}|}_{SS}\\ & C_{L}^{Fe^{3+}}\leq {C_{L}^{Fe^{3+}}|}_{SS} \end{array}$$ (42)

The two expressions for *R*^*IHepc*^ are designed to allow *R*^*IHepc*^ to be continuous across the range of liver iron concentration. At steady state,

$$R^{IHepc}=k_{IHepc,Fe^{3+}}\left({\left({C_{L}^{Fe^{3+}}|}_{SS}\right)}^{n_{IHepc,Fe^{3+}}}/\left({\left({C_{L}^{Fe^{3+}}|}_{SS}\right)}^{n_{IHepc,Fe^{3+}}}+{\left(km_{IHepc,Fe^{3+}}\right)}^{n_{IHepc,Fe^{3+}}}\right)\right)$$ (43)

## Methods

Values for all initial model variables and parameters are listed in tables described later in the document. From the literature, we specified steady-state reference values for initial basic model variables and parameters. To reduce the number of parameters to be estimated, we assumed relationships between species transport parameters based on molecular weight *MW* of each species such as:

$$\frac{h_{ISF-P}^{j}}{h_{ISF-P}^{Tf}}=\sqrt{\frac{MW_{Tf}}{MW_{j}}};\;\frac{h_{P\to EB}^{j}}{h_{P\to EB}^{Tf}}=\sqrt{\frac{MW_{Tf}}{MW_{j}}}$$ (44)

The steady-state concentration of iron hemoglobin (*HbFe*) in plasma represents typical values of healthy adult male individuals [2] assuming that at least 95% of the total iron hemoglobin is bound to iron. Some parameter values were computed from steady-state relationships among the variables (as indicated in the Table 5) and some parameter values were obtained directly from literature (as indicated in the Table 6). Some initial conditions of the model were also obtained by using simple steady-state relationships.

**Table 5:** Steady-state relationships between parameters.

Parameter	Relationship
*V*_*P*_	*V*_*B*_(1−*hct*)
*v*_0_	$v_{cfu,ss}\frac{\left(C_{P,ss}^{Epo}+Km_{Epo}\right)}{C_{P,ss}^{Epo}}$
$C_{X}^{O_{2}}$ *X* = [*I*,*M*,*ISF*]	$\frac{P_{O_{2}}}{\sigma_{O_{2}}}$
$X_{CFU}^{TfR}$	*N*_*TfR*0_/(*Avg*_no*1*E*+9,*Avg*_*no* = 6.023*E* + 23
$C_{EB,ss}^{TfR}$	$\frac{F_{CFU\to EB}^{ss}*X_{CFU}^{TfR}}{V_{EB}*k_{RBC\leftarrow EB}}$

**Table 6:** Parameter values obtained from literature.

Parameter name	Description	Units	Value
*α*_1_	Rate coefficient for the linear time based change in volume of plasma after phlebotomy [25, 52]	min^−1^	3.76E-5
*α*_2_	Rate coefficient for the exponential time based change in volume of plasma after phlebotomy [25, 52]	min^−1^	8.43E-5
*β*_*P*_	Fraction of plasma volume removed due to phlebotomy [25, 52]	dimensionless	8.00E-2
*β*_*CFU*_	Rate coefficient for rate of proliferation of CFUe with age [25, 52]	min^−1^	1.44E-3
*hct*_0_	Baseline healthy serum hematocrit [53]	dimensionless	4.60E-1
*HbFe*_*P*,*SS*_	Baseline healthy serum concentration of serum iron hemoglobin [53]	*μM*	9.39E+3
$k_{Cp(Cu^{1+}),O_{2}}$	Rate of oxidation of the Cu^1+^ to Cu^2+^ in ceruloplasmin by O_2_ [22]	*μM*^−1^ min^−1^	5.93E+0
$k_{Cp(Cu^{2+}),Fe^{2+}}$	Rate coefficient of oxidation of Fe^2+^ to Fe^3+^ by ceruloplasmin [22]	*μM*^−1^ min^−1^	8.99E+1
$k_{Fe^{2+},Fe^{3+}}$	Rate coefficient of conversion of Fe^3+^ to Fe^2+^ [22]	min^−1^	1.88E+0
$k_{Fe^{3+},Fe^{2+}}$	Rate coefficient of conversion of Fe^2+^ to Fe^3+^[22]	min^−1^	3.19E-2
$k_{Fe^{2+},FPN}$	Rate coefficient of binding of Fe^2+^ to intracellular FPN [22]	*μM*^−1^ min^−1^	6.30E-1
$k_{Fe^{2+},O_{2}}$	Rate coefficient of oxidation of Fe^2+^ to Fe^3+^ by O_2_ [22]	*μM*^−1^ min^−1^	6.24E+0
$k_{Fe^{3+}FPN,Fe^{3+}}$	Rate coefficient of release of Fe^3+^ from (Fe^3+^)FPN complex [22]	min^−1^	1.99E+0
$k_{DTfR{\left(Fe^{3+}\right)}_{2}Tf}$	Rate coefficient of dissociation of (Fe^3+^)_2_Tf from complex with TfR [54]	*μM*	8.46E-3
$k_{DTfR(Fe^{3+})Tf}$	Rate coefficient of dissociation of (Fe^3+^)Tf from complex with TfR [54]	*μM*	3.38E-2
*MW*_*Tf*_	Molecular weight of transferrin [55]	kDa	7.00E+1
$MW_{FeSO_{4}}$	Molecular weight of FeSO_4_ [56]	Da	1.52E+2
*MW*_*Cp*_	Molecular weight of ceruloplasmin [57]	kDa	1.32E+2
*MW*_*Fe*_	Molecular weight of elemental iron	Da	5.6E+1
*MW*_*Hepc*_	Molecular weight of hepcidin [58]	Da	2.70E+3
*MW*_*HbFe*_	Molecular weight of iron hemoglobin [59]	kDa	1.66E+3
*μ*_*F*_	Age at which CFUe mature into erythroblasts [6]	min	5.76E+3
$\sigma_{O_{2}}$	Solubility coefficient for O_2_ in medium/serum [6]	L*atm / *μmole*	7.69E-4
$C_{Hepc,SS}^{P}$	Steady-state concentration of hepcidin in serum [14]	*μM*	1.50E-2
*V*_*I*_	Volume of the intracellular compartment of the RES compartment [22]	*mL*	1.00E-3
*V*_*M*_	Volume of the membrane compartment of the RES compartment [22]	*mL*	1.00E-4
*τ*_*Tf*_	Half-life of transferrin in serum [6, 53, 60]	min	1.152E+4
$C_{P}^{Tf_{total}}$	Total concentration of serum transferrin [54, 61]	*mg*/*dL*	2.81E+2
*N*_*TfR*0_	Number density of transferrin receptors on erythroblasts [62, 63]	*receptor* / *cell*	4.00E+5
$n_{IHepc,Fe^{3+}}$	Hill function’s coefficient for increase in hepcidin expression by Fe^3+^ [20]	dimensionless	2

### Transferrin in plasma

The steady-state concentrations of the three transferrin species *Tf*_*P*_,(*Fe*^3+^)*Tf*_*P*_,(*Fe*^3+^)_2_*Tf*_*P*_ are related by stoichiometry [4] as:

$$C_{P}^{(Fe^{3+})Tf}+C_{p}^{{\left(Fe^{3+}\right)}_{2}Tf}+C_{P}^{Tf}=C_{P}^{Tf_{total}}$$ (45)

From experiments [2, 64], $C_{P}^{Tf_{total}}=40\mu M$ is the mean total serum transferrin. Based on the mean serum transferrin saturation of 50% in healthy male subjects [1, 2], we can write:

$$\frac{2C_{p}^{{\left(Fe^{3+}\right)}_{2}Tf}+C_{P}^{(Fe^{3+})Tf}}{2C_{P}^{Tf_{total}}}=0.5$$ (46)

The measured relative concentrations of these species [65, 66] is approximately:

$$\frac{C_{P}^{(Fe^{3+})Tf}}{2}=\frac{C_{p}^{{\left(Fe^{3+}\right)}_{2}Tf}}{1}=\frac{C_{P}^{Tf}}{1}$$ (47)

The initial (and steady-state) values of *Tf*_*j*_,(*Fe*^3+^)*Tf*_*j*_,(*Fe*^3+^)_2_*Tf*_*j*_ in other regions (*j* = *ISF*, *EB*) are assumed equal to the corresponding values in plasma.

Parameters of the model were estimated in stages with estimates from the previous step carried forward for future parameter estimation steps. Some model parameters were estimated by using small parts of the model to experimental data e.g. the kinetics of transferrin to TFR, binding of iron (*Fe*^3+^) to *Tf* and *Fe*^3+^*Tf*.

### Transferrin receptor-transferrin complex

From *in vitro* experiments [67], the normalized concentration (y) of I^125^-labelled (*Fe*^3+^)_2_*Tf* that is internalized can be described by

$$y=\frac{C_{TfR{\left(Fe^{3+}\right)}_{2}Tf}}{C_{TfR{\left(Fe^{3+}\right)}_{2}Tf}^{0}}=1-e^{-k_{recycle,TfR}t}$$ (48)

where $C_{TfR{\left(Fe^{3+}\right)}_{2}Tf}^{0}$ is the initial concentration of the complex and *k*_*recycle*,*TfR*_ is the rate constant for internalization and recycling. The value of this parameter was obtained by fitting the model to the data as shown in Supplement S2 Fig.

### Transferrin binding to transferrin receptor

To estimate the differential binding rates of mono- and di-ferric transferrin to transferrin receptor ($k_{TfR,(Fe^{3+})Tf},k_{TfR,{\left(Fe^{3+}\right)}_{2}Tf}$), we used *in vitro* cell-culture data [40, 65–67]. These data show time-course interaction of mono- and di-ferric transferrin to transferrin receptors over a wide range of doses. The corresponding model equations describe mono-ferric transferrin ((*Fe*^3+^)*Tf*) and diferric transferrin ((*Fe*^3+^)_2_*Tf*) binding to free transferrin receptor (*TfR*):

$$\begin{array}{l} \frac{dC_{(Fe^{3+})Tf}}{dt}=-k_{onTfR(Fe^{3+})Tf}C_{(Fe^{3+})Tf}C_{TfR}+k_{offTfR(Fe^{3+})Tf}C_{TfR(Fe^{3+})Tf}\\ \frac{dC_{{\left(Fe^{3+}\right)}_{2}Tf}}{dt}=-k_{onTfR{\left(Fe^{3+}\right)}_{2}Tf}C_{{\left(Fe^{3+}\right)}_{2}Tf}C_{TfR}+k_{offTfR{\left(Fe^{3+}\right)}_{2}Tf}C_{TfR{\left(Fe^{3+}\right)}_{2}Tf}\\ \frac{dC_{TfR(Fe^{3+})Tf}}{dt}=k_{onTfR(Fe^{3+})Tf}C_{(Fe^{3+})Tf}C_{TfR}-k_{offTfR(Fe^{3+})Tf}C_{TfR(Fe^{3+})Tf}-k_{recycle}C_{TfR(Fe^{3+})Tf}\\ \frac{dC_{TfR{\left(Fe^{3+}\right)}_{2}Tf}}{dt}=k_{onTfR{\left(Fe^{3+}\right)}_{2}Tf}C_{{\left(Fe^{3+}\right)}_{2}Tf}C_{TfR}-k_{offTfR{\left(Fe^{3+}\right)}_{2}Tf}C_{TfR{\left(Fe^{3+}\right)}_{2}Tf}-k_{recycle}C_{TfR{\left(Fe^{3+}\right)}_{2}Tf}\\ C_{TfR}+C_{TfR(Fe^{3+})Tf}+C_{TfR{\left(Fe^{3+}\right)}_{2}Tf}=C_{TfR0} \end{array}$$ (49)

where *C*_*TfR*0_ is the concentration of free transferrin receptors in the absence of any transferrin.

By definition, the binding parameters are related as:

$$\begin{array}{l} k_{offTfR(Fe^{3+})Tf}=k_{onTfR(Fe^{3+})Tf}k_{DTfR(Fe^{3+})Tf}\\ k_{offTfR{\left(Fe^{3+}\right)}_{2}Tf}=k_{onTfR{\left(Fe^{3+}\right)}_{2}Tf}k_{DTfR{\left(Fe^{3+}\right)}_{2}Tf} \end{array}$$ (50)

The relative affinities of (*Fe*^3+^)*Tf* and (*Fe*^3+^)_2_*Tf* are known (Table 6). The unknown parameters in the model $k_{recycle},k_{onTfR(Fe^{3+})Tf},k_{onTfR{\left(Fe^{3+}\right)}_{2}Tf},C_{TfR0}$ are estimated by matching the model output of iron bound to transferrin with different starting concentrations of mono-ferric and diferric transferrin [65–67] as shown in S3 Fig.

### Iron binding with transferrin in plasma

In previous work [22], the rates of binding of *Fe*^3+^ to *Tf* and *Fe*^3+^*Tf* were assumed to be the same. However, the rate constants for binding of *Fe*^3+^*Tf* and (*Fe*^3+^)_2_*Tf* to *TfR* are different (as described above) so that the plasma concentration of *Fe*^3+^*Tf* is more than that of (*Fe*^3+^)_2_*Tf*. To achieve the expected concentrations of *Fe*^3+^*Tf* and (*Fe*^3+^)_2_*Tf* in blood at steady-state, the rates of binding for *Fe*^3+^ and *Fe*^3+^*Tf* had to be different.

The next step was to estimate some unknown parameters of the *in vivo* model by matching the simulated output to the steady-state concentrations of the known species. For example, the model parameters $h_{ISF\to P}^{j},h_{P\to EB}^{j},k_{EB,Hb,Fe},k_{Fe^{3+},Tf},k_{Fe^{3+},Fe^{3+}Tf}$ were estimated by matching the model outputs to steady-state values of clinical markers. As described above, distinctive values of binding rate constants were obtained for *Fe*^3+^*Tf* and (*Fe*^3+^)_2_*Tf* to *TfR* and these were used in the model simulations during the parameter estimation process. The remaining parameters were estimated by comparing model outputs in response to perturbations corresponding to experimental time-course data. These perturbation experiments were carefully chosen to estimate parameters in small sets. The best parameter estimates are those that minimize the least-squared difference between the model outputs and experimental data. In order to develop confidence that the parameter estimates are global, a Differential Evolution algorithm [68] was used with multiple restarts to find the best result.

Model parameters were estimated using experimental data associated with the following perturbations applied to the basic model:

1. Phlebotomy produces a loss of blood volume that leads to changes in serum hemoglobin and Epo concentrations. This experiment allows calibration of changes in Epo synthesis and secretion in response to changes in serum hemoglobin.
2. Iron ingestion increases serum iron levels and Hepc synthesis leading to increased serum Hepc. This experiment allows calibration of the dose response of Hepc synthesis and secretion in response to changes in serum iron and the rate of degradation of FPN by Hepc.
3. rhHepc injection changes serum Hepc concentration that reduces FPN and inhibits iron transport leading to drop in serum iron. This experiment performed in mice allows estimation of the half-life of FPN which is crucial for simulation of long-term changes in FPN levels *in vivo*. Differences in model parameters between mouse and human are listed in Table 7.
4. rEpo injection affects CFUe dynamics leading to increased hemoglobin in blood.
5. Anemia of CKD leads to loss of sensitivity of Epo synthesis to changes in serum hemoglobin and decrease in baseline Epo synthesis.

**Table 7:** Parameter values from literature different between human and mouse model.

Parameter name	Description	Units	Value (human)	Value (mice)
*V*_*B*_	Volume of plasma compartment	*mL*	5.04E+2	3.00E+0
*V*_*EB*_	Volume of erythroblast compartment	*mL*	1.89E+3	2.54E+0
*V*_*ISF*_	Volume of the ISF compartment	*mL*	4.29E+2	1.95E-1
*τ*_*RBC*_	Half-life of RBC in plasma	*day*	1.20E+2	4.00E+1
*τ*_*CFU*_	Half-life of CFUe	min	5.76E+3	2.88E+3
*τ*_*EB*_	Half-life of erythroblasts	min	2.88E+3	1.44E+3

Optimal estimates of model parameters were obtained by least-square fitting of model outputs under a variety of conditions to experimental data. Simulation of model outputs requires numerical solution of model equations. Steady-state values of the model outputs are obtained by solving the model equations for a sufficiently long time until the output values change negligibly. The model consists of differential and algebraic equations. To convert the partial differential equation (Eq 7) into this format, we discretized spatial derivatives [69]. *(Code available as supplementary material)*. The model equations are solved as an initial-value problem using a Python code based on LSODES [70]. LSODES was specifically used to numerical solve the stiff differential equations because the biological system has variables which change over different time scales.

### System perturbations for parameter estimation

From the transient model, a steady state is reached by simulating the model for a long time. This provides initial conditions for the following perturbations:

#### Phlebotomy

Standard phlebotomy or blood loss occurs with an 8% reduction of blood volume, which leads to dilution of both blood cells and protein concentrations. Over several hours, the intravascular volume is replenished by the slow movement of fluid from the interstitial space into blood leading to dilution of both blood cells and proteins. The release of iron from stores is necessary to simulate the time course of recovery of after phlebotomy. For this simulation, we modified the basic model equations by incorporating equations based on previous studies [25].

As a consequence of blood loss, the plasma volume decrease is represented as:

$$\frac{dV_{P}}{dt}=-\alpha_{2}V_{P0}\left(\alpha_{1}t-\beta_{P}\right)e^{-\alpha_{2}t}$$ (51)

which upon integration yields:

$$V_{P}=V_{P0}\left(1+\left(\alpha_{1}t-\beta_{P}\right)e^{-\alpha_{2}t}\right)\;\Rightarrow \;V_{P}(0)=V_{P0}\left(1-\beta_{P}\right)$$ (52)

where reflects initial blood loss and are empirical rate parameters [25]. Accounting for plasma volume loss, the plasma concentration for species j that depends on diffusion fluxes between phases changes as:

$$\frac{dC_{P}^{j}}{dt}=J_{ISF\to P}^{j}-J_{P\to EB}^{j}-\frac{C_{P}^{j}}{V_{P}}\frac{dV_{P}}{dt}$$ (53)

where

$$\frac{1}{V_{P}}\frac{dV_{P}}{dt}=\frac{-\alpha_{2}\left(\alpha_{1}t-\beta_{P}\right)e^{-\alpha_{2}t}}{1+\left(\alpha_{1}t-\beta_{P}\right)e^{-\alpha_{2}t}}$$ (54)

Compared to the basic model equations, the modified plasma concentrations involve this loss in plasma volume, which tends to increase concentration with time. The modified equations for Epo and Hepc are

$$\frac{dC_{P}^{Epo}}{dt}=T_{K\to P}^{Epo}-\frac{C_{P}^{Epo}}{\tau_{}^{Epo}}-\frac{C_{P}^{Epo}}{V_{P}}\frac{dV_{P}}{dt},\;\frac{dC_{P}^{Hepc}}{dt}=T_{L\to P}^{Hepc}-\frac{C_{P}^{Hepc}}{\tau_{}^{Hepc}}-\frac{C_{P}^{Hepc}}{V_{P}}\frac{dV_{P}}{dt}$$ (55)

Blood loss leads to release of iron from stores in the liver, which is reflected in the equation representing the *Fe*^*3+*^ concentration change:

$$V_{P}\frac{dC_{P}^{Fe^{3+}}}{dt}=J_{ISF\to P}^{Fe^{3+}}-J_{P\to EB}^{Fe^{3+}}-C_{P}^{Fe^{3+}}\frac{dV_{P}}{dt}+T_{L\to P}^{Fe^{3+}}$$ (56)

The rate of iron release is controlled by Hepc according to the empirical relation:

$$T_{L\to P}^{Fe^{3+}}=\min\left[T_{L0}^{Fe^{3+}}[1-\eta_{Hepc}^{Fe^{3+}}(C_{Hepc}^{SS}-C_{Hepc})],0\right]$$ (57)

where $\eta_{Hepc}^{Fe^{3+}}$ are empirical parameters. Also, blood volume change affects the RBC number density:

$$\frac{dN_{RBC}}{dt}=\frac{k_{RBC\leftarrow EB}}{V_{P}}N_{EB}-d_{RES\leftarrow RBC}N_{RBC}-\frac{N_{RBC}}{V_{P}}\frac{dV_{P}}{dt}$$ (58)

The dynamic model outputs (basic model including the phlebotomy perturbation) are fit to time course data of serum concentrations of hemoglobin () and Epo () from literature [25] to obtain optimal estimation of parameters that affect Epo synthesis, CFUe maturation, Hepc, and iron release from stores.

#### Iron ingestion

After ingestion, iron is transported from gut into plasma. The rate of transport can be approximated as a 1^st^ order process and expressed as:

$$T_{Gut\to P}^{Fe^{3+}}=k_{Gut\to P}Y_{0}^{Fe^{3+}}\exp(-k_{Gut\to P}t)$$ (59)

where $Y_{0}^{Fe^{3+}}$ represents the oral iron dose and *k*_*Gut*→*P*_ is the rate coefficient of absorption. With this perturbation including plasma volume reduction, the concentration of plasma iron changes as:

$$\frac{dC_{P}^{Fe^{3+}}}{dt}=\frac{J_{ISF\to P}^{Fe^{3+}}-J_{P\to EB}^{Fe^{3+}}}{V_{P}}+T_{Gut\to P}^{Fe^{3+}}$$ (60)

The model output incorporating this perturbation was fit to experimental time-course data for serum iron ($C_{P}^{Fe}$), transferrin saturation ($X_{P}^{Tf}$) and serum hepcidin ($C_{P}^{Hepc}$) [11]. The serum iron and transferrin saturation are defined as:

$$C_{P}^{Fe}=\left(C_{P}^{(Fe^{3+})Tf}+2C_{P}^{{\left(Fe^{3+}\right)}_{2}Tf}\right)*MW_{Fe}/10$$ (61)

$$X_{P}^{Tf}=\frac{(C_{P}^{(Fe^{3+})Tf})/2+C_{P}^{{\left(Fe^{3+}\right)}_{2}Tf}}{C_{P}^{Tf}+C_{P}^{(Fe^{3+})Tf}+C_{P}^{{\left(Fe^{3+}\right)}_{2}Tf}}$$ (62)

*MW*_*Fe*_ represents the molecular weight of elemental iron.

#### Drug injection

The perturbations associated with injections of rhHepc and rEpo require model equations for drug concentrations in plasma and in a generic tissue. For this purpose, a 2-compartment pharmacokinetics model is applied (S1 Fig). Drug (*j* = *rhHepc*, *rEpo*) concentration in the plasma compartment changes according to

$$\frac{dC_{P}^{j}}{dt}=\frac{k_{P\leftarrow T}C_{T}^{j}-k_{T\leftarrow P}C_{P}^{j}}{V_{P}}-\frac{C_{P}^{j}}{\tau_{P}^{j}}+T_{EL}^{j}$$ (63)

where *k*_*P*←*T*_ and *k*_*T*←*P*_ are rate coefficients for transport between plasma (*P*) and tissue (*T*) and

$\tau_{P}^{j}$ is the time constant of drug loss by several processes including metabolism. The rate of drug entry per plasma volume, which depends on the entry location, is represented as:

$$T_{EL}^{j}=k_{EL}Y_{EL}^{0}e^{-k_{EL}t}$$ (64)

where *EL* = *Peri*,*SC*,*Gut*,*k*_*EL*_ is the first-order rate of drug entry and $Y_{EL}^{0}$ is the dose of the drug injected. The drug concentration in tissue changes as:

$$\frac{dC_{T}^{j}}{dt}=-\frac{k_{P\leftarrow T}C_{T}^{j}-k_{T\leftarrow P}C_{P}^{j}}{V_{T}}$$ (65)

#### Injection of rhHepc

With this perturbation, *rhHepc* acts like endogenous Hepc and causes degradation of FPN in the intracellular and membrane regions of the RES compartment. This is indicated by a dashed line in Fig 2 and represented by an additional reaction:

FPN+rhHepc→⊗ (66)

The basic model is modified by incorporating the drug injection equations. To estimate the model parameters, the dynamic model output was fit to the time course of serum drug *(rhHepc)* concentration (S4 Fig). In the *rhHepc* study, the drug was injected into mice through the peritoneum [71]. Mice data were used because data are not available from human studies. Consequently, there were changes in volumes and several other factors as described in Table 7.

#### Injection of rEpo.

With this perturbation, rEpo acts like endogenous Epo and increases the rate of BFUe differentiation into CFUe as represented by

$$N_{CFU}(0,t)=\left\{ \begin{array}{l} N_{BFUe}\left[\left(\frac{C_{P}^{Epo}(t)}{C_{P}^{Epo}(0)}\right)+\left(\frac{\alpha_{N_{BFU}}^{rEpo}C_{P}^{rEpo}}{C_{P}^{rEpo}+EC_{50}^{rEpo}}\right)\right]\;\mathrm{for}C_{P}^{Epo}(t)<C_{P}^{Epo}(0)\\ N_{BFUe}\left(\frac{\alpha_{N_{BFU}}^{rEpo}C_{P}^{rEpo}}{C_{P}^{rEpo}+EC_{50}^{rEpo}}\right)\;\mathrm{for}C_{P}^{Epo}(t)\geq C_{P}^{Epo}(0) \end{array}\right.$$ (67)

The model parameters $\alpha_{N_{BFU}}^{rEpo}$ and $EC_{50}^{rEpo}$ represent the sensitivity of BFUe to rEpo and the half-maximum concentration for the drug. Another effect of adding *rEpo* is to increase the rate of CFUe maturation as represented by

$$v_{CFU}=v_{0}\left(\frac{C_{P}^{Epo}}{C_{P}^{Epo}+Km_{Epo}}\right)+\left(\frac{\alpha_{v_{CFU}}^{rEpo}C_{P}^{rEpo}}{C_{P}^{rEpo}+EC_{50}^{rEpo}}\right)$$ (68)

The model parameter $\alpha_{v_{CFU}}^{rEpo}$ represents the sensitivity of CFUe maturation to rEpo concentrations.

To estimate the model parameters listed in Table 8, the dynamic model output is fit to the time course of serum drug *(rEpo)* concentration. In the *rEpo* study, the drug is administered both as intravenous (IV) infusion or subcutaneous injection in humans.

**Table 8:** Estimated parameters for pharmacokinetics and pharmacodynamics of rEpo.

Parameter name	Description	Units	Value (CV%)
*k*_*P*→*T*,*rEpo*_	Rate coefficient for transport of rEpo from plasma to tissue compartment	mL*min^−1^	2.84E+0 (12.1)
*K*_*T*→*P*,*rEpo*_	Rate coefficient for transport of rEpo from tissue to plasma compartment	mL*min^−1^	7.52E+0 (22.4)
*V*_max,*rEpo*_	Rate coefficient for the maximal rate of metabolism of rEpo	mM*min^−1^	1.22E-1 (10.7)
*τ*_*rEpo*_	Half-life of rEpo	min	2.27E+2 (8.3)
$EC_{50}^{rEpo}$	Half-maximal concentration of rEpo for effect on CFUe and erythroblasts	*mM*	2.97E+2 (34.2)
$\alpha_{N_{BFU}}^{rEpo}$	Rate coefficient for increase in BFU entering the maturation cycle due to rEpo	dimensionless	1.83E-1 (23.1)
$\alpha_{v_{CFU}}^{rEpo}$	Rate coefficient for increase in maturation rate of CFUe due to rEpo	dimensionless	3.37E-1 (15.9)

## Results

We applied our mechanistic model of iron metabolism to quantitatively analyze differences in the status of iron metabolism in chronic kidney disease (CKD) with anemia before and after treatment. The model simulates a treatment strategy for CKD anemia with rEpo injections and iron-dextran infusion. The effect of the treatment protocol on the status of iron metabolism, especially the iron fluxes, is quantified by our model.

### Phlebotomy responses

After phlebotomy, the time course of Epo concentration in plasma and in hematocrit over 60 days has been measured [25]. For comparison with data, hematocrit was evaluated from model-simulated RBC and plasma volumes:

$$hct(t)=\frac{V_{RBC}(t)}{V_{RBC}(t)+V_{P}(t)};\;V_{RBC}(t)=\left(V_{Blood,SS}-V_{P,SS}\right)\left(\frac{N_{RBC}(t)}{N_{RBC}^{SS}}\right)$$ (69)

**Fig 3:**
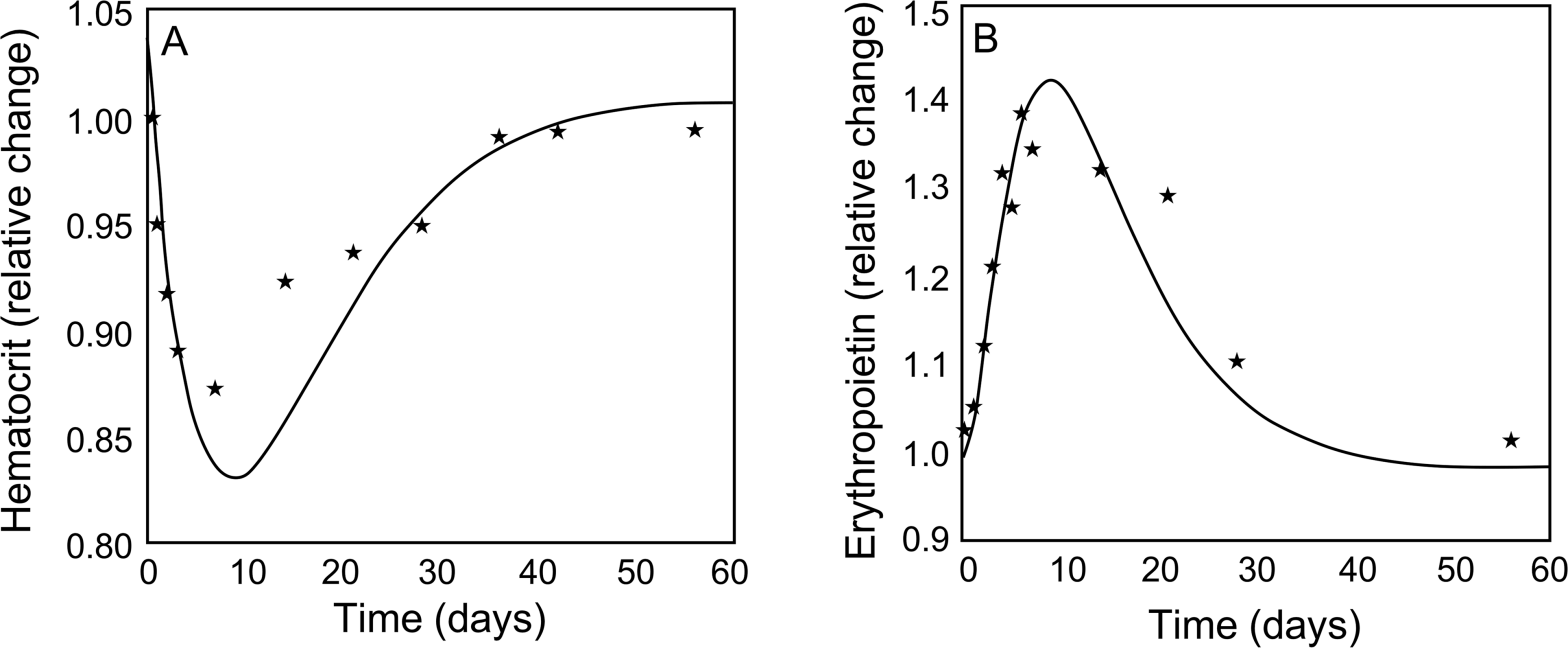
Comparison of model output (solid line) to experimental data (*) of relative changes in (A) hematocrit and (B) erythropoietin concentration in plasma for 60 days in response to phlebotomy.

The data for hematocrit and Epo in plasma were normalized to initial values to compensate for differences in steady-state values. Matching model outputs to these data, the model parameters (*α*^*Epo*^,*β*^*Epo*^,*τ*^*Epo*^, *Km*_*Epo*_) were estimated (as indicated in Table 9). Simulated responses to phlebotomy (Fig 3A & 3B) follow trends of the experimental data [25]. The hematocrit decreases for the first ten days and rebounds to the initial level over the next 50 days (Fig 3A), while the time course of Epo concentration is the opposite (Fig 3B).

**Table 9:** Parameter values estimated based on experimental data.

Parameter name	Description	Units	Value (CV%)
$k_{TfR,Fe^{3+}Tf}$	Rate coefficient for binding of mono-transferrin to transferrin receptor	*μM*^−1^min^−1^	1.63E-1 (5.2)
$k_{TfR,{\left(Fe^{3+}\right)}_{2}Tf}$	Rate coefficient for binding of holotransferrin to transferrin receptor	*μM*^−1^min^−1^	7.67E-1 (3.5)
*k*_*recycle*,*TfR*_	Rate coefficient for recycling of endocytosed transferrin-transferrin receptor complex	min^−1^	6.83E-1 (2.1)
$h_{P\to EB}^{Tf}$	Rate coefficient for transport of transferrin from plasma to EB compartment	mL*min^−1^	9.01E+0 (11.2)
$h_{P\to ISF}^{Fe^{3+}}$	Rate coefficient for transport of iron from plasma to ISF compartment	mL*min^−1^	1.25E-2 (15.2)
$h_{P\to ISF}^{Tf}$	Rate coefficient for transport of transferrin from plasma to ISF compartment	mL*min^−1^	1.26E+2 (11.4)
$h_{I\to M}^{Fe^{3+}}$	Rate coefficient for transport of iron from intracellular to membrane compartment inside the RES compartment	mL*min^−1^	1.76E+0 (17.8)
$k_{Fe^{3+},Tf}$	Rate coefficient for binding of Fe^3+^ to apo-transferrin	*μM*^−1^min^−1^	2.87E-1 (5.6)
$k_{Fe^{3+},FeTf}$	Rate coefficient for binding of Fe^3+^ to mono-transferrin	*μM*^−1^min^−1^	3.16e-2 (4.5)
$k_{Fe^{3+},Hb}$	Rate coefficient for binding of Fe^3+^ to hemoglobin	*μM*^−1^min^−1^	4.35E+2 (43.8)
*α*^*Epo*^	Rate coefficient for increase in Epo synthesis due to change in serum hemoglobin	dimensionless	4.04E-1 (16.7)
*β*^*Epo*^	Scaling factor for exponential increase in Epo synthesis due to change in serum hemoglobin	*μM*^−1^	4.26E-1 (22.1)
*τ*_*Epo*_	Half-life of Epo in plasma	Min	6.66E+2 (17.9)
*Km*_*Epo*_	Half-maximal concentration of Epo for effect on erythroblasts	*μM*	6.00E+0
*τ*_*EPN*_	Half-life of ferroportin in the RES compartment	min	3.06E+4 (34.2)
*k*_*RES*,*Hepc*,*FPN*_	Rate coefficient of removal of FPN by Hepc	*μM*^−1^min^−1^	4.39E-1 (21.5)
*k*_*RES*,*rhHepc*,*FPN*_	Rate coefficient of removal of FPN by rhHepc	*μM*^−1^min^−1^	1.19E-1 (12.3)
$k_{TfR2,Fe^{3+}Tf}$	Rate coefficient for binding of mono-ferric transferrin to TfR2	*μM*^−1^min^−1^	2.56E-2 (5.2)
$k_{TfR2,{\left(Fe^{3+}\right)}_{2}Tf}$	Rate coefficient for binding of diferric transferrin to TFR2	*μM*^−1^min^−1^	5.42E-2 (3.5)
$k_{IHepc,Fe^{3+}}$	Rate coefficient for expression of IHepc by Fe^3+^	μM*min^−1^	2.87E-1 (34.1)
$km_{IHepc,Fe^{3+}}$	Half-maximal concentration of Fe^3+^ for expression of IHepc	*μM*	4.51E+2 (45.2)
$kI_{IHepc,Fe^{3+}}$	Half-maximal inhibitory concentration of Fe^3+^ for expression of IHepc	*μM*	4.01E-2 (11.3)
$k_{Gut\to P}^{Fe^{3+}}$	Rate coefficient for transport of orally administered iron into plasma	μmoles*min^−1^	2.77E-2 (4.2)
*τ*_*IHepc*_	Half-life of IHepc	min	7.41E+1 (42.9)
*τ*_*Hepc*_	Half-life of Hepc in plasma	min	4.37+2 (33.1)
$d_{P}^{Fe^{3+}}$	Rate of removal of iron from plasma	min^−1^	6.93E+0 (29.3)

### Responses to rhHepc injection

**Fig 4:**
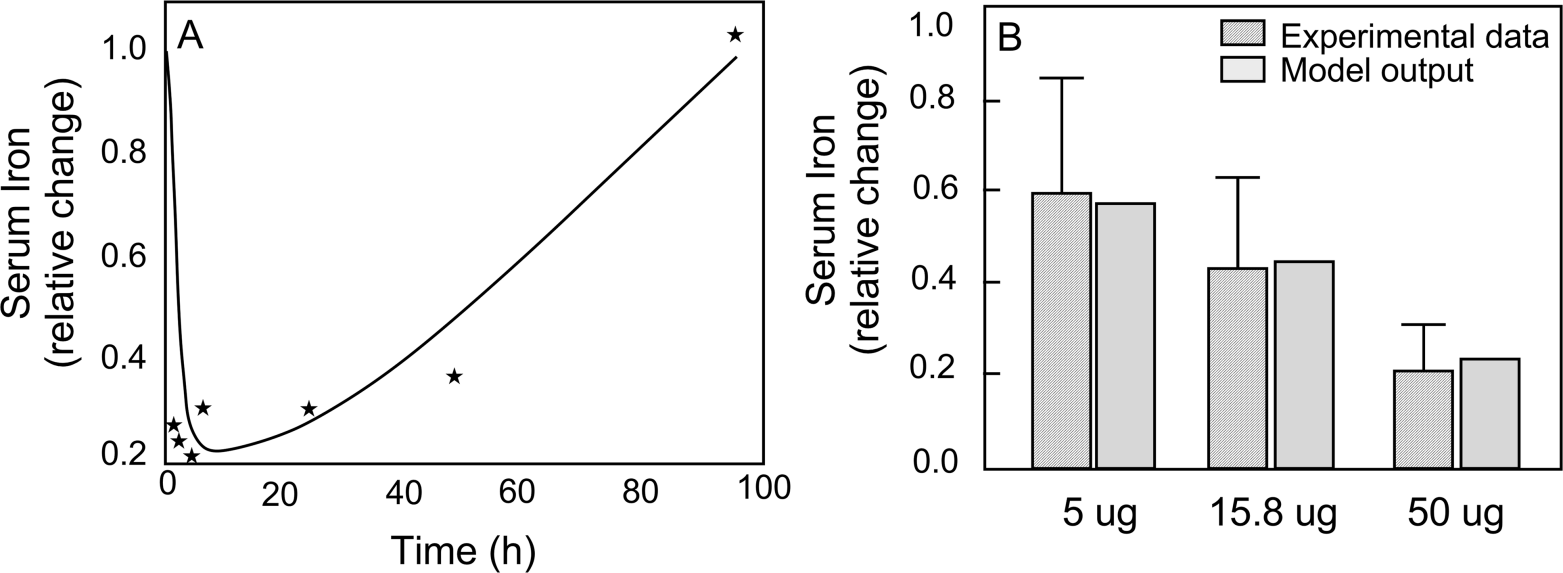
Comparison of model-simulated serum iron to experimental data in mouse after rhHepc injection (A) Time course after injection of 50 μg of rhHepc; solid line is the model output and (*) represent the experimental data (B) maximum change of serum iron with different doses of rhHepc.

In a mouse model of iron metabolism [71], changes of rhHepc and serum iron were measured after injection of 50, 15.8, and 5.0 *μg* rhHepc. The first step was estimation of model parameter for the pharmacokinetics of rhHepc in mice (as indicated in Table 10, S4 Fig). The metabolic model parameters (*k*_*RES*,*rhHepc*,*FPN*,_*τ*_*EPN*_) were estimated (Table 9) by matching serum iron output using the pharmacokinetic model for rhHepc in conjunction with the whole-body model to the normalized serum iron data. The time course of serum iron simulates data after injection of 50 *μg* of rhHepc (Fig 4A). Within 5 h, serum iron is reduced by 80%, but gradually returns to the initial value about 100 h after the injection (Fig 4A). The model also predicts the maximum changes in serum iron after injection of different doses of rhHepc (Fig 4B).

**Table 10:** Estimated parameter for mouse pharmacokinetics of rhHepc.

Parameter name	Description	Units	Value (CV%)
*k*_*P*→*T*,*rhHepc*_	Rate coefficient for transport of rhHepc from plasma to tissue compartment	mL*min^−1^	4.70E-2 (23.8)
*k*_*T*→*P*,*rhHepc*_	Rate coefficient for transport of rhHepc from tissue to plasma compartment	mL*min^−1^	3.71E-2 (31.2)
*τ*_*rhHepc*_	Half-life of rhHepc in plasma	min	4.13E+1 (34.1)

### Responses to iron ingestion

**Fig 5:**
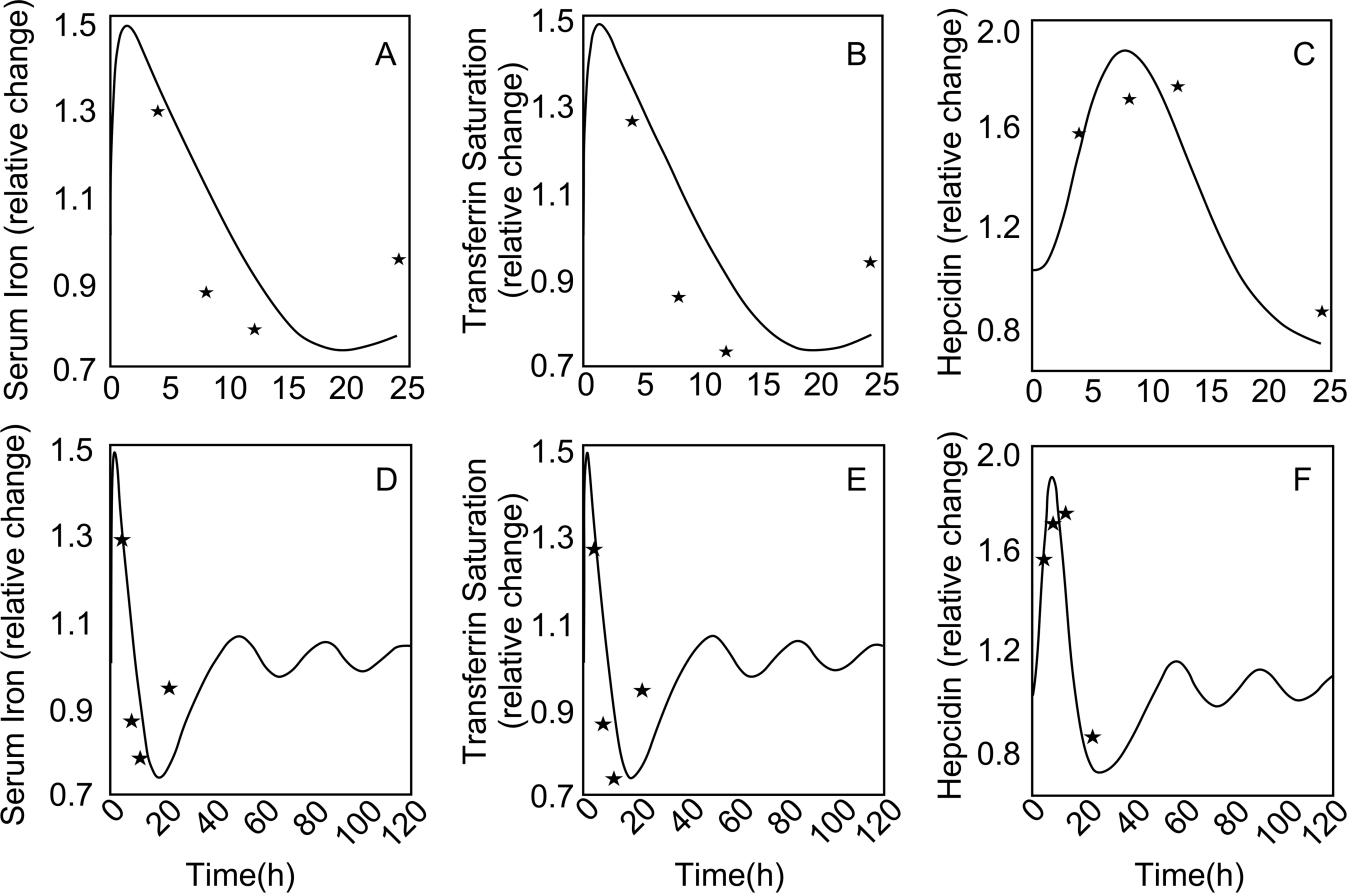
Comparison of simulated (solid line) responses to iron ingestion to experimental data (*) for a short period of 2h past iron ingestion—(A) Serum iron (B) Transferrin saturation (C) Hepcidin concentration. The naturally damped oscillations produced as a result are shown by simulation of a longer time period (5 days) for the same variables (D-F).

Simulations of the iron ingestion experiment conducted on healthy human subjects by Girelli et al [50] incorporate all parameter values estimated via different perturbations and experiments (e.g. the half-life of FPN etc.) including steady-state relationships, mouse rhHepc injection and phlebotomy experiment. The data for serum iron, serum transferrin saturation and serum hepcidin were normalized with their initial, steady-state values. From these data, we obtained optimal parameter estimates ($d_{P}^{Fe^{3+}},k_{Gut\to P}^{Fe^{3+}},k_{RES,Hepc,FPN}$ $\tau_{Hepc},\tau_{IHepc},k_{IHepc,Fe^{3+}},km_{IHepc,Fe^{3+}},kI_{IHepc,Fe^{3+}}$) (Table 9). Following ingestion of iron, the model simulates the specific time courses of serum iron and transferrin saturation that go up and down together (Fig 5A and 5B), but also the time course of serum Hepc concentration which also shows an increase and decrease from steady-state but delayed in time as compared to serum iron (Fig 5C). Model simulations of the iron ingestion experiment for an extended period (5 days) are also presented to emphasize the oscillatory behavior for all species (Fig 5D–5F)

### Responses of RBC (or Hb) to rEpo

For treatment of CKD anemia, rEpo (epoietin-alpha) is administered regularly and repeatedly. The dynamics of rEpo concentrations were simulated using a PK model for both intravenous and subcutaneous administration of rEpo as above (S6A–S6C Fig). The optimal values of $\alpha_{N_{BFU}}^{rEpo}$, $EC_{50}^{rEpo}$, $\alpha_{v_{CFU}}^{rEpo}$ (Table 8) are those that provided the best simulation of hematocrit data (S5 Fig) from healthy subjects during repeated IV injections (100 IU/kg) of rEpo over 4 weeks [72, 73]. The model incorporates a constraint such that serum hemoglobin ($HbFe_{P}^{3+}$) reaches saturation with long-term repeated doses of rEpo (S5 Fig).

### Effects of CKD with anemia and iron treatments

**Fig 6:**
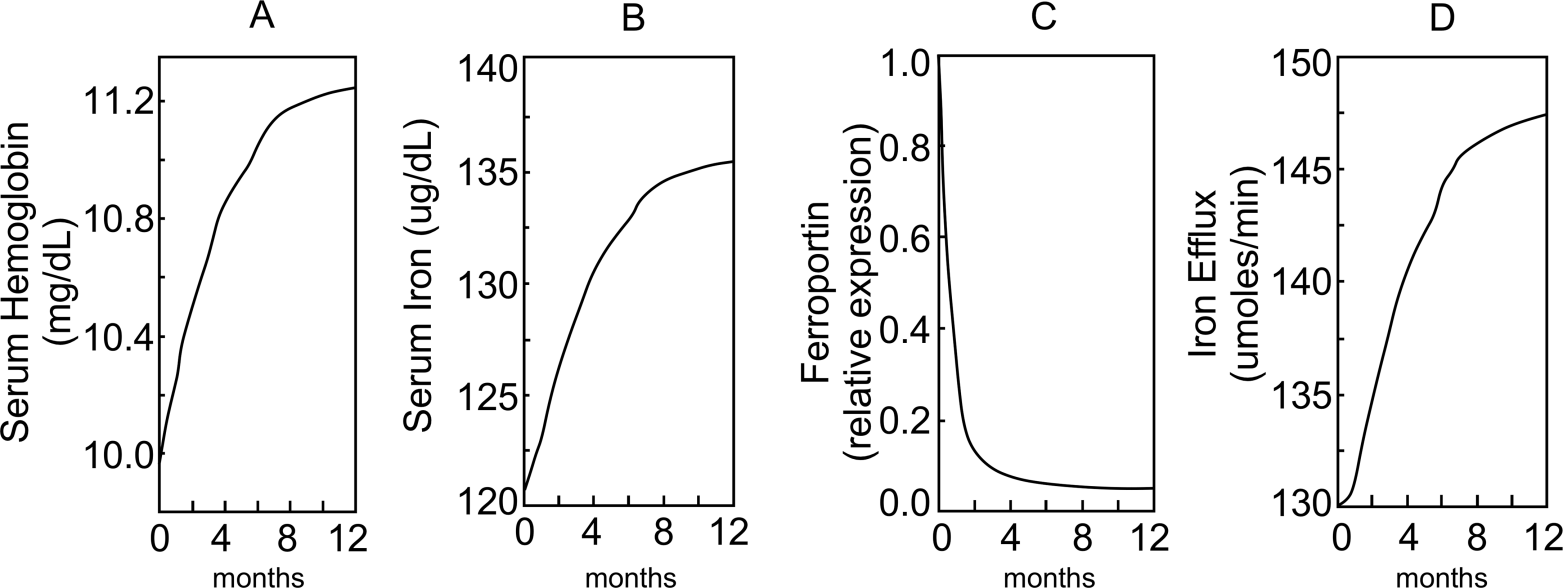
Model simulation to show the variation in (A) serum iron-hemoglobin (mg/dL), (B) serum iron (μg/dL), (C) intracellular ferroportin (FPN), (D) efflux of iron from RES (μmoles/min), when a CKD patient is treated with combination of epoietin-alpha (rEpo = 100 μg/Kg) and IV Iron Dextran (1g) every 2 weeks for 12 months. The time course of change of all four variables over time have been plotted from the start of the treatment at time 0.

Our model simulates the effects of different levels of CKD anemia on plasma RBC number density due to reduced levels of serum Epo (S7 Fig) over 2 years. In these simulations, the initial concentration of plasma Epo ($C_{0}^{Epo}$) varied between 6 pM (normal) and 3.6 pM (severe anemia). This allows the RBC number density to reach 50% of normal (S7A Fig) and serum hemoglobin to reach 9.8 mg/dL (S7B Fig). Similar drops are observed in serum iron (S7C Fig) and transferrin saturation (S7D Fig). Most of the decrease in RBC number density occurs within a year. Simulated treatments of CDK anemia for patients with a starting Hb~10 mg/dL, $C_{0}^{Epo}$ ≤ 4.6 pM and *α*^*Epo*^ = 0 are shown in Fig 6. The simulated treatment consists of epoietin-alpha injections (rEpo = 100 ug/Kg) every 2 weeks and IV iron-dextran (1g) every 2 weeks for 12 months. The treatment regimen is obtained based on published guidelines and recent literature [74–78]. For these simulations, changes in two serum markers–hemoglobin (A) and serum iron (B) are shown along with two measures–ferroportin levels in cells of the RES (C) and iron efflux from RES (D), which cannot be easily measured *in vivo*.

As explained before, all simulations of the whole-body model, especially the perturbations, start from a set of steady-state which is obtained by simulating the model for a period of time and allowing it to reach a steady-state. This steady-state solution for each of the species is described below in Table 11.

**Table 11:** Initial values of all species.

Variable name	Value (*μM*)
$C_{P}^{Tf}$	1.38E+1
$C_{P}^{Fe^{3+}Tf}$	2.12E+1
$C_{P}^{{\left(Fe^{3+}\right)}_{2}Tf}$	5.85E+0
$C_{P}^{Fe^{3+}}$	0
$C_{P}^{Epo}$	6.0E+0
$C_{P}^{IHepc}$	9.99E-3 (dimensionless)
$C_{P}^{Hepc}$	1.5E-2
$C_{P}^{HbFe^{3+}}$	1.66E+4
$C_{P}^{Tf}$	1.38E+1
$C_{EB}^{TfR}$	8.81E-1
$C_{EB}^{TfRFe^{3+}Tf}$	4.02E-1
$C_{EB}^{TfR{\left(Fe^{3+}\right)}_{2}Tf}$	1.03E-1
$C_{EB}^{Fe^{3+}Tf}$	1.93E+0
$C_{EB}^{{\left(Fe^{3+}\right)}_{2}Tf}$	1.05E-1
$C_{EB}^{Fe^{3+}}$	7.98E-6
$C_{EB}^{Tf}$	3.80E+1
$C_{EB}^{Hb}$	1.04E+2
$C_{EB}^{HbFe^{3+}}$	2.40E+3
$C_{I}^{Fe^{2+}}$	6.76E-1
$C_{I}^{Fe^{3+}}$	3.03E+1
$C_{I}^{FPN}$	3.79E+0
$C_{I}^{Fe^{2+}FPN}$	1.05e-5
$C_{M}^{Fe^{3+}}$	1.87e-6
$C_{M}^{FPN}$	1.05e-5
$C_{M}^{Fe^{2+}FPN}$	2.33e-2
$C_{M}^{Fe^{3+}FPN}$	1.64e+0
$C_{M}^{Fe^{3+}}$	1.87e-5
$C_{M}^{Fe^{3+}Tf}$	2.25E+1
$C_{M}^{{\left(Fe^{3+}\right)}_{2}Tf}$	5.41E+1
$C_{M}^{Tf}$	1.21E+1
$C_{M}^{Cp(Cu^{2+})}$	2.27E+0
$C_{M}^{Cp(Cu^{1+})}$	3.39E-4
$C_{ISF}^{Fe^{3+}Tf}$	2.25E+1
$C_{ISF}^{{\left(Fe^{3+}\right)}_{2}Tf}$	5.41E+0
$C_{ISF}^{Tf}$	2.27E+0
$C_{ISF}^{Fe^{3+}}$	1.46E-1
$C_{ISF}^{Cp(Cu^{2+})}$	2.27E+0
$C_{ISF}^{Cp(Cu^{1+})}$	3.18E-4
$C_{L}^{TfR2}$	1.53E+0
$C_{L}^{TfR2Fe^{3+}Tf}$	1.63E-1
$C_{L}^{TfR2{\left(Fe^{3+}\right)}_{2}Tf}$	7.74E-2
$C_{L}^{Fe^{3+}Tf}$	1.34E+1
$C_{L}^{{\left(Fe^{3+}\right)}_{2}Tf}$	1.35E+0
$C_{L}^{Fe^{3+}}$	4.33E+1

## Discussion

### Mechanistic mathematical model for analysis

Our multi-scale model of iron metabolism integrates molecular mechanisms with cellular and tissue transport of iron in organ systems of the whole body (Figs 1 & 2). A top-down modeling strategy was applied to incorporate only enough mechanistic and empirical structure (Fig 2) to reliably simulate key features in the experimental data. Furthermore, the model was used to predict key physiological responses that have not been measured while maintaining the biological consistency of the underlying processes. All expressions in the model are based on causal mechanistic understanding of processes and not based on associations or correlations observed in observed data. Examples of key model mechanisms includes transferrin receptor-mediated uptake of iron in erythroblasts and ferroportin-mediated iron release from RES. This model does not assume a simple gradient approach to all transport processes, which is common in previous models of whole-body iron metabolism [18, 19, 39]. Furthermore, it integrates data from *in vitro* cellular experiments, mouse experiments, healthy human volunteer studies, and clinical studies of anemia with chronic kidney disease (CKD). The model also incorporates the pharmacokinetics of different drugs (rhHepc, rEpo) and the effects downstream of the changing drug concentrations. This has not been done in previous whole-body iron metabolism models. Of specific clinical value is the model simulation of anemia in CKD patients with insufficient erythropoietin and treatments with rEpo and iron dextran infusion. This model was calibrated using a variety of experimental mouse and human data.

In developing this model, we tried to balance mechanistic detail with limitations imposed by available data. The goal is to relate every expression in the model to some realistic abstraction of a physiological or biological process such that the model parameters and responses can then help explain and quantify the underlying behavior. An example of this strategy relates to modeling the secretion of Hepc based on serum iron. While Hepc is made in the liver, the regulation of Hepc synthesis is downstream of transferrin receptor 2 and regulated by an intricate network of intracellular signaling pathways which involve a variety of other molecules, e.g., HFE [51]. Applying a few key reasonable assumptions, the model for regulation of Hepc synthesis can be simplified and still maintain biological integrity to simulate and predict the dynamics and dose response characteristics observed. The goal is to add enough detail to represent the dose response and dynamics of Hepc to changes in serum iron, which is different from previous models [21]. To predict the dynamics of iron release in the RES, detailed mechanisms that describe the interaction between serum iron, Hepc and iron release are essential. Previous models of whole-body iron metabolism [18, 19, 79, 80] are limited by not differentiating the different forms of iron (Fe^2+^, Fe^3+^) and by not incorporating appropriately the roles of ferroportin in iron release and Hepc in iron homeostasis. Most previous models assume ferroportin acts as a pore on the cell surface with passive diffusion of iron rather than facilitated transport [22]. This model distinguishes the different iron forms, mechanistic roles of ferroportin and hepcidin, and incorporates enzymes (e.g., ceruloplasmin) in iron metabolism. The iron stores in the I-region of the RES compartment represent the overall iron stores in the model. While the model does not account for total ferritin stores in the human body, Fe^3+^ is the closest proxy for ferritin stores in the model, though it likely underestimates the total ferritin content.

Our strategy for this study was to develop a model with the smallest number of compartments and minimum molecular detail that can reproduce experimental data and provide a mechanistic basis for prediction. For example, the CFU section is assumed spatially distributed because a simpler spatially lumped model cannot reproduce the expected responses. However, the RES compartment uses a verified model with more molecular detail than is needed for this application. However, this model is well tested and can be used in future studies of iron deficiency anemia due to ceruloplasmin deficiency, mutations or even copper deficiency.

Once the model structure was established, the values of model parameters were estimated based on experimental data from the literature. A step-wise qualitative sensitivity analysis determined the type of experimental data needed to evaluate system parameters. Enough experimental data was available to constrain the model parameters for analysis of the CKD anemia treatment. To obtain optimal estimates of the parameters for the various subsystems, we simulated a step-wise sequence of responses to phlebotomy, rhHepc injection, iron ingestion, and rEpo injections and a small set of parameters was estimated using each of the simulations and all parameters from previous steps were carried forward. The reliability of the estimated parameters was estimated by calculating the coefficient of variation (CV) for each parameter for the specific simulation scenario of the estimation process. The CV for most estimated parameters was around or below 10%. Furthermore, the cross-correlation coefficients were estimated, but no significant cross-correlation was observed and hence not reported.

### Model assumptions and limitations

The model assumes that iron recycling is 100%. While most of iron is recycled (>95%), the loss of iron is usually replenished by uptake of iron from diet [4, 24, 53]. This dietary uptake of iron is tightly controlled by Hepc. The uptake of iron from the gut lumen to blood also involves more than one transporter [4] and the details of iron uptake from the gut are not included in this model. Gut mediated iron uptake becomes significant especially in anemia of CKD patients for whom oral iron therapy is generally ineffective. Such details maybe further refined in the model in future iterations.

Iron is stored in the liver and in the RES as ferritin. When plasma iron is deficient, ferritin acts as a temporary store of iron. Ferritin is not included as a separate species in the model. However, Fe^3+^ in the intracellular compartment of RES is a modest proxy for ferritin in the system. Furthermore, the model considers the RES system of the liver and spleen (macrophages) as a single functional compartment (RES); hence the iron stores are lumped into the RES compartment and no separate iron transport in the liver is considered. During model simulation of iron deficiency (e.g. phlebotomy) there is release of iron from the Fe^3+^ stores of the I-region of the RES compartment. Similarly, during iron overload (e.g. iron dextran treatment in CKD anemia) the Fe^3+^ levels increase significantly in the model. The iron in the liver compartment (L) is solely used for control of hepcidin synthesis in the model. Hepatocytes in the liver also contain *TfR1* receptors, which is yet another means of iron uptake from serum transferrin. This iron can also be released through ferroportin on the cells. Control of expression of iron in the hepatocytes has been incorporated in the model through the *TfR2* pathway, but not through the *TfR1* pathway. This will affect the dynamics of iron transferrin in the serum, delivery of iron to CFUe and hepcidin expression. The model also does not incorporate the expression of erythroferrone by the erythroblasts and its inhibitory effect on hepcidin synthesis [81]. These limitations can be incorporated in a future version of the model as more data becomes available.

The whole-body model incorporates a model of iron release from monocytes [22] based on *in vitro* iron release experiments from U937 cells, which is similar, but not the same as iron release *in vivo* in mice and human. Using the data from iron sequestration by rhHepc injection experiments and model simulation, the half-life of ferroportin (protein) is estimated. Information about the half-life of ferroportin protein levels is unknown, but significant in design of experimental studies involving iron release regulation and Hepc. The only information available is the half-life of ferroportin mRNA, but that is known to be significantly smaller than that of the stable transporter protein. This model estimate of ferroportin protein half-life is the first reported and should be validated using properly designed experiments. The value of ferroportin half-life is critical in all model simulations which involve degradation of FPN due the effect of increase in Hepc levels, e.g., iron ingestion and iron dextran administration in CKD patients with anemia, and gradual recovery of FPN levels over time.

CKD patients have been reported to have blood loss [82]. This has not been incorporated into the model currently. This can potentially change the concentrations of all serum species. However, in the simulation of CKD, the serum concentration of Epo is calibrated to achieve specific levels of serum hemoglobin. While, the levels of hemoglobin are still correct for CKD patients, it is likely that the levels of Epo needed to achieve them in the model could vary from those observed in CKD patients.

### Application and limitations of experimental data

With this global model involving a large number of variables and parameters and limited experimental data, the model validation process cannot be exhaustive. The time course data of hematocrit and Epo concentrations in response to phlebotomy (Fig 4A and 4B) were obtained by combining data from different experiments [25]. Since the observations were not from a single experiment, the variability in the combined data are expected to be greater than normal. Nevertheless, the model simulations correspond well to the data. For this simulation, the model parameter values estimated were related to the dose response of erythropoiesis to Epo concentration and the serum half-life of Epo.

To mathematically model iron metabolism in the mouse using information from human iron metabolism, it is necessary to scale the compartmental volumes and RBC half-life in blood. The RBC half-life, which is 4 times less in mice [83], has a great effect on serum iron when rhHepc is injected. The model simulation of rhHepc injection (Fig 5A) shows that the serum iron drops to its minimum around 8hr and then gradually rises back to normal around 96hr. However, the mouse experimental data shows that the serum iron drops to a near minimum in nearly 1hr and does not change for 8hr. This feature cannot be explained with the current model.

In response to iron ingestion, model simulations show oscillatory behavior with respect to serum iron (Fig 5A) and transferrin saturation (Fig 5B). This is not evident from the Hepc response (Fig 5C) because the maximum value of hepcidin occurs about 600 min after the maximum value of serum iron. However, when the model is allowed to simulate 4 days (more than the 1 day from experimental data), the oscillations in serum iron and Hepc are seen (Fig 5D–5F). Such oscillations have significant impact on iron release from the RES in response to perturbations in iron metabolism through disease or treatment. From a modeling perspective, the appearance of an oscillation suggests a higher-order system. The model produces this naturally by the combination of Hill’s function type response and feedback loops related to regulation of serum iron by hepcidin associated with changes in iron release from RES and changes of hepcidin secretion associated with serum iron.

Due to a lack of experimental data, the model could not be exhaustively validated. Additional experimental data that can significantly improve the model & provide robust validation would include (1) time-course data over a range of doses of iron ingested rather than a single dose (2) time-course data on individual patients with CKD anemia receiving treatment, and (3) measurement of iron stores and iron flux through animal models of CKD anemia.

### Analysis of CKD anemia and treatment

Reduced levels of serum Epo leads to reduced levels of RBC number density and serum hemoglobin in a dose response manner (S7 Fig). In CKD patients, it has been observed that serum levels of Hepc are often higher than normal even with reduced serum iron [84]. This is explained by the role of inflammation on Hepc synthesis [85]. The current model does not support inflammation in CKD patients or its role in Hepc synthesis. Thus, in this model, Hepc levels are lower than normal in CKD patients. Model simulations of treatment of CKD anemia with rEpo and iron-dextran, show the improvement in RBC number density (not shown), serum hemoglobin (Fig 6A) and serum iron (Fig 6B). The effect of the treatment is gradual as can be seen through comparison of the results from 1 month vs. 12 months of treatment (Fig 6A and 6B). The injection of iron-dextran into the system was carefully simulated to avoid a sudden, large increase in serum iron as reported [33]. However, even with a slow increase in serum iron, the model simulations predict that serum Hepc increased substantially (not shown), which over time caused nearly complete degradation of intracellular ferroportin in the macrophages of the RES (Fig 6C). According to the model simulations, iron release from the RES system is increased only by 20% during the 12 months of simulation (Fig 6D). Investigations into the fate of all the iron injected revealed that the levels of iron in the RES system that were 50% of steady-state levels at the start of the treatment increased by 3.8 fold in 1 month and 15.1 fold above control in 12 months. The model simulations highlight that while the current treatment is able to improve the serum hemoglobin levels, it is not fully utilizing the recycling process characterizing iron metabolism. The significant accumulation of iron in the RES system is likely to have additional pathological effects [36, 37]. Because the current model does not take into account uptake of iron in hepatocytes through the *TfR1* pathway, accumulation of iron during such treatment in hepatocytes cannot be simulated. Then it would be expected that the estimates of accumulation of iron in the RES would be reduced by the amount accumulated in the hepatocytes. Also, the current model is missing the negative regulation of hepcidin expression mediated through erythroferrone, which could be significant due to rhEPO administration.

### Single vs ensemble predictions

In the simulation of CKD with treatments, only the best calibrated model was used for all simulations. Ideally, one could generate an ensemble of models generated by varying all the parameters estimated around the best estimated values. The large ensemble of models can then be compared to the training data across all the scenarios using likelihood-based scores. Based on these likelihood scores, predictions from each of the individual models can be combined to generate a mean prediction and confidence interval (CI) for the predictions. This approach requires knowledge of the error rates for each of the different types of measurements used in likelihood scoring and subsequent large-scale simulations. The total number of parameters estimated in this model is high, so the requirement of the ensemble size would be very large. This makes the generation of ensemble predictions intractable for the scope of this study and has been avoided.

### Conclusion and future studies

This multi-scale, whole-body model of iron metabolism not only simulates data from a wide range of experimental studies, but also predicts novel responses that have not been observed. Future versions of the model may be developed to add further mechanistic detail to iron absorption in the gut and the regulation of hepcidin synthesis as shown in some studies [21]. Of special clinical significance are the side effects associated with the CKD anemia treatment by rEpo and iron-dextran, which are related mechanistically to iron transport and pathways of iron metabolism. Future studies with this model could analyze (a) hepcidin increase as a defensin in patients with CKD that exacerbates anemia [86] or (b) the effect of increased levels of inflammatory load of cytokines in patients with CKD anemia that causes the RBC lifespan to decrease [73, 86, 87]. Beyond the effects and mechanisms of CKD anemia, other aspects associated with iron metabolism could be investigated using this model as a platform for the analysis of copper deficiency on iron metabolism through ceruloplasmin or even other iron metabolism related genetic mutations.

## Supporting information

S1 Fig Pharmacokinetic model incorporating absorption, distribution and elimination of rhHepc and rEpo. P represents drugs taken orally, SC represents subcutaneous administration, V_T_ represents the volume of the tissue compartment.
(TIF)

S2 Fig Comparison of the model output (solid line) and experimental data (*) for the recycling of transferrin receptors from the surface. (TIF)

S3 Fig Comparison of the model output (hashed boxes) vs experimental data (solid boxes) for binding of iron transferrin to transferrin receptors on erythroblasts for varying concentration of iron (11–90 μg/dL) measured at 60 min or 120 min as denoted. (TIF)

S4 Fig Comparison of the model output (solid line) vs. experimental data (*) for changes in concentration of rhHepc in mice after a single peritoneal injection of 50μg of rhHepc. (TIF)

S5 Fig Comparison of model simulation (solid line) and experimental data (*) for relative changes in hematocrit in response to rEpo injections (3/wk) over 4 weeks. (TIF)

S6 Fig Comparison of model simulation (solid line) against experimental data (*) of serum concentration over time of rEpo with different doses of rEpo injections (A) IV 50IU/Kg (B) 50 IU/Kg SC and (C) 100 IU/kg IV.
(TIF)

S7 Fig Represents model simulation of development of anemia of chronic kidney disease. Serum erythropoietin is set to decrease from 6pM to 3.6pM at increments of 0.6pM to represent increasing severity of CKD and the model outputs are shown for a period of 2 yrs. The model can reproduce varying degrees of anemia of CKD as shown with the reduction in plasma red blood cell density (A), plasma hemoglobin (B), serum iron (C) and transferrin saturation (D).
(TIF)

S1 Data (GZ)
