# Supplementary figures and images for: Whole-body iron transport and metabolism: Mechanistic, multi-scale model to improve treatment of anemia in chronic kidney disease
Source: PLoS Comput Biol. 2018 Apr 16;14(4):e1006060. doi: 10.1371/journal.pcbi.1006060 (PMC5919696; doi:10.1371/journal.pcbi.1006060)

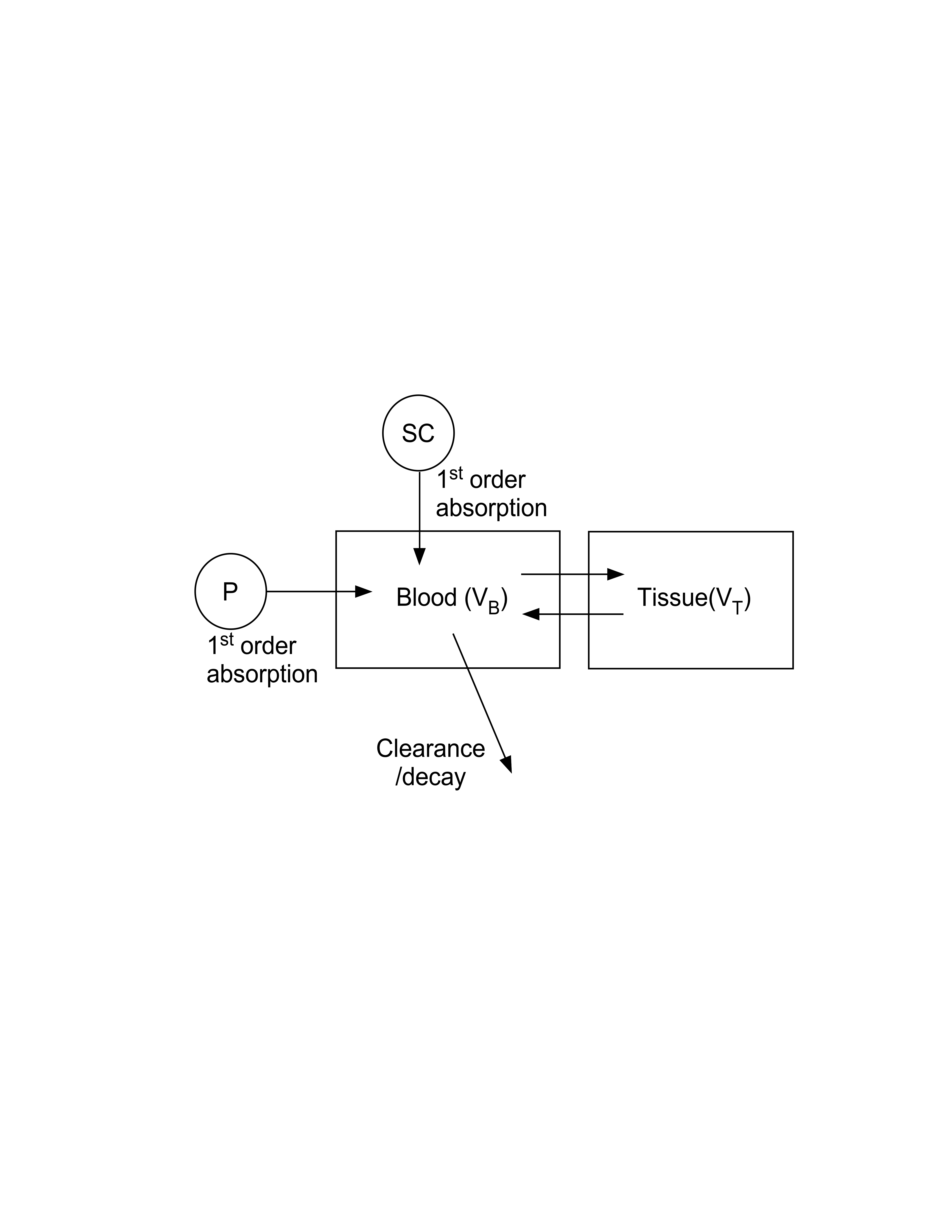

Supplement: S1 Fig — P represents drugs taken orally, SC represents subcutaneous administration, VT represents the volume of the tissue compartment. (TIF) [file pcbi.1006060.s001.tif]

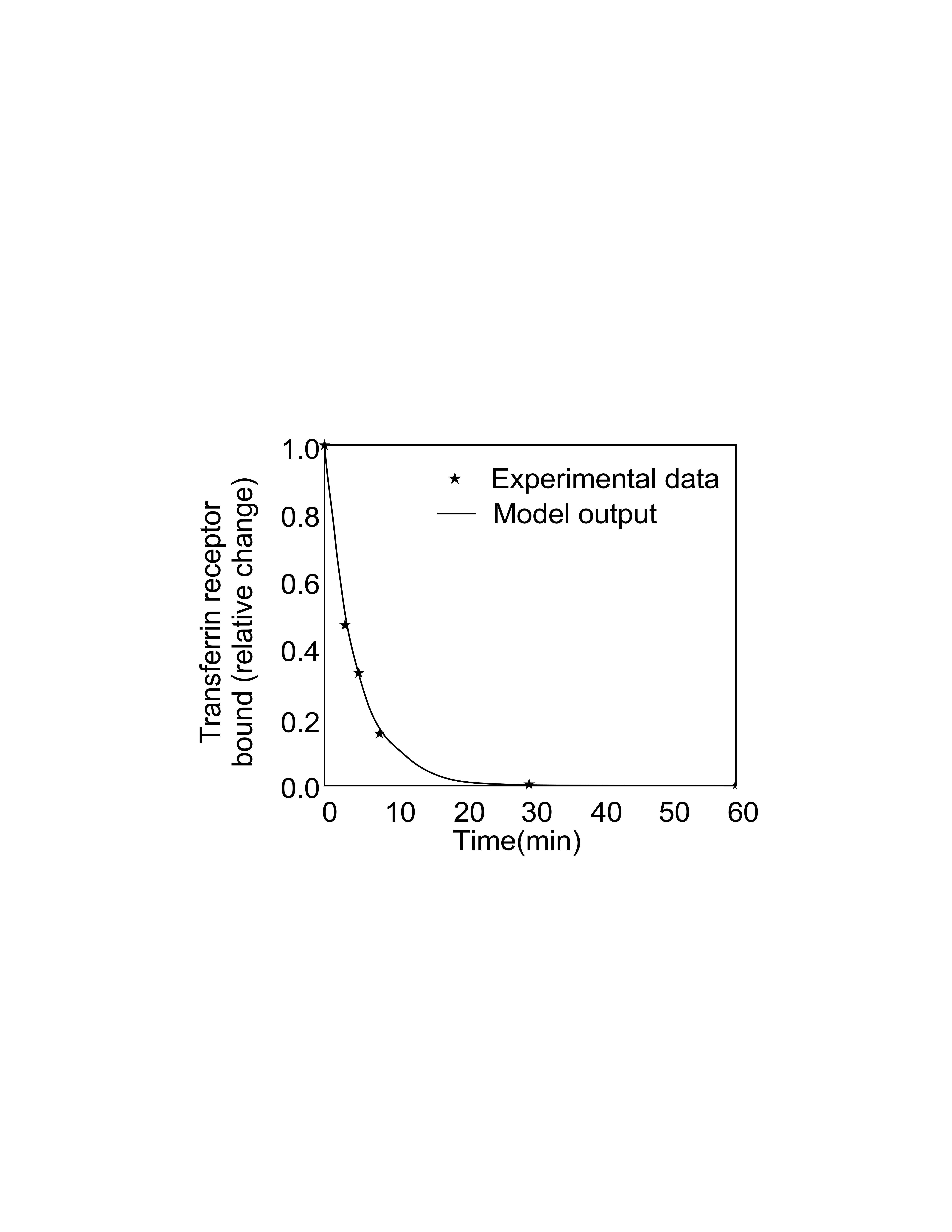

Supplement: S2 Fig — (TIF) [file pcbi.1006060.s002.tif]

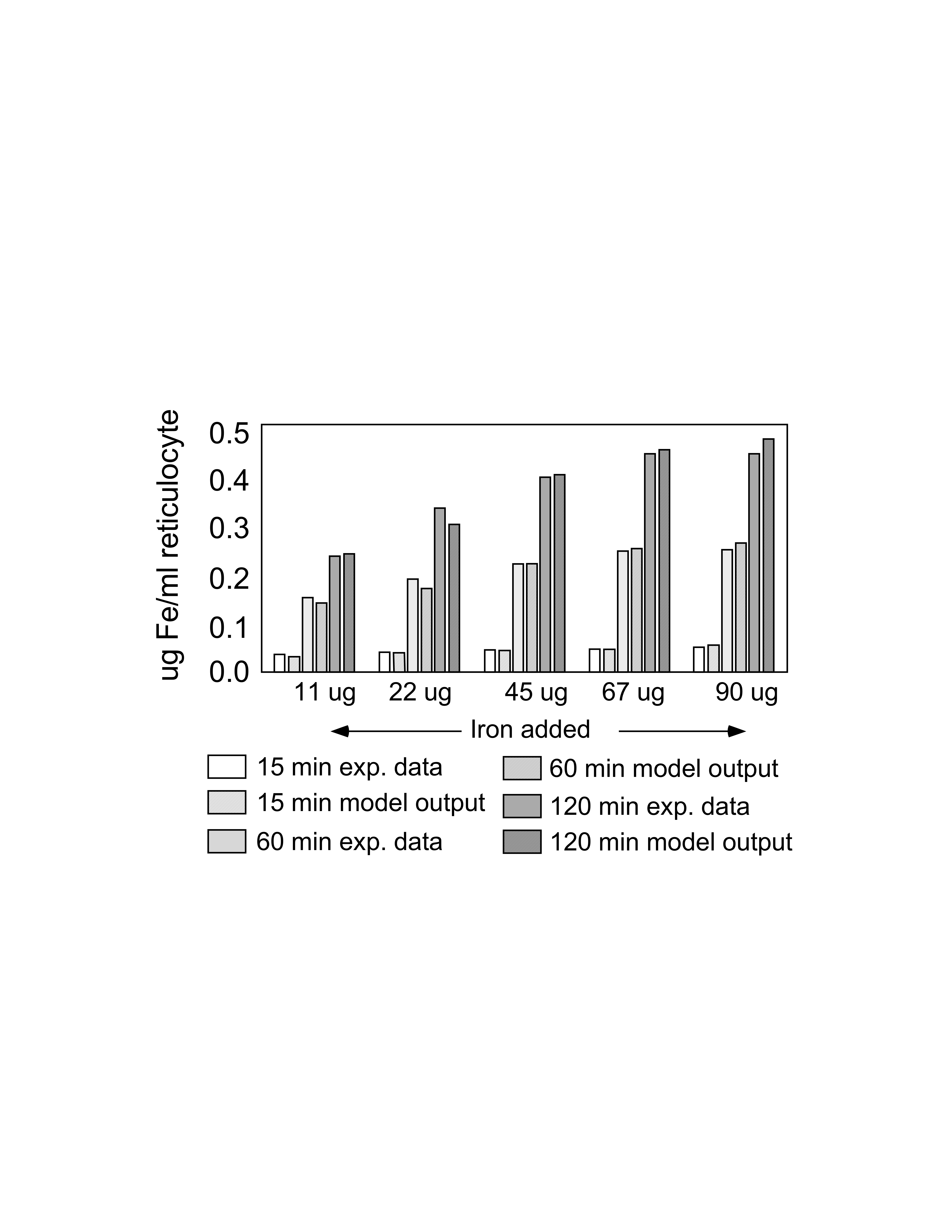

Supplement: S3 Fig — (TIF) [file pcbi.1006060.s003.tif]

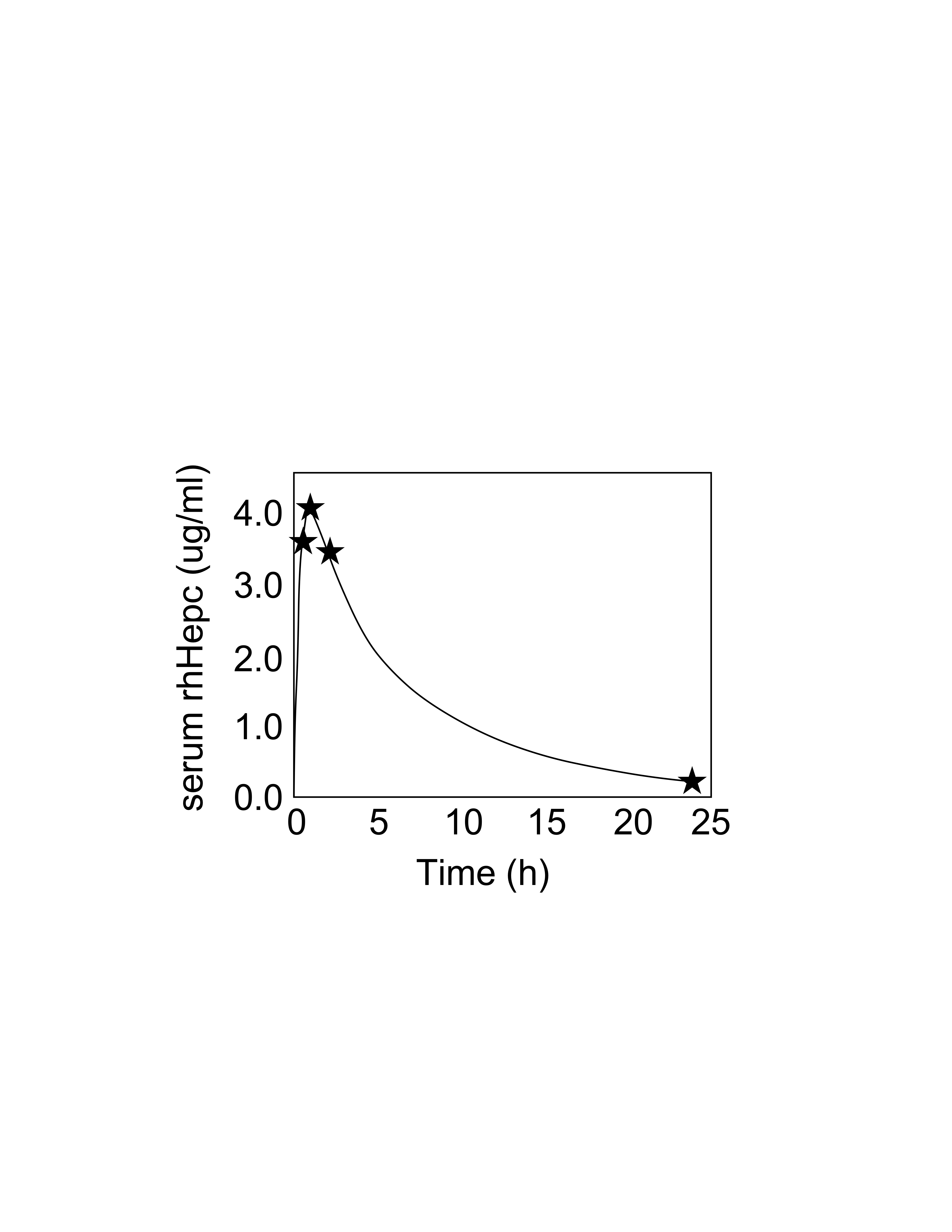

Supplement: S4 Fig — (TIF) [file pcbi.1006060.s004.tif]

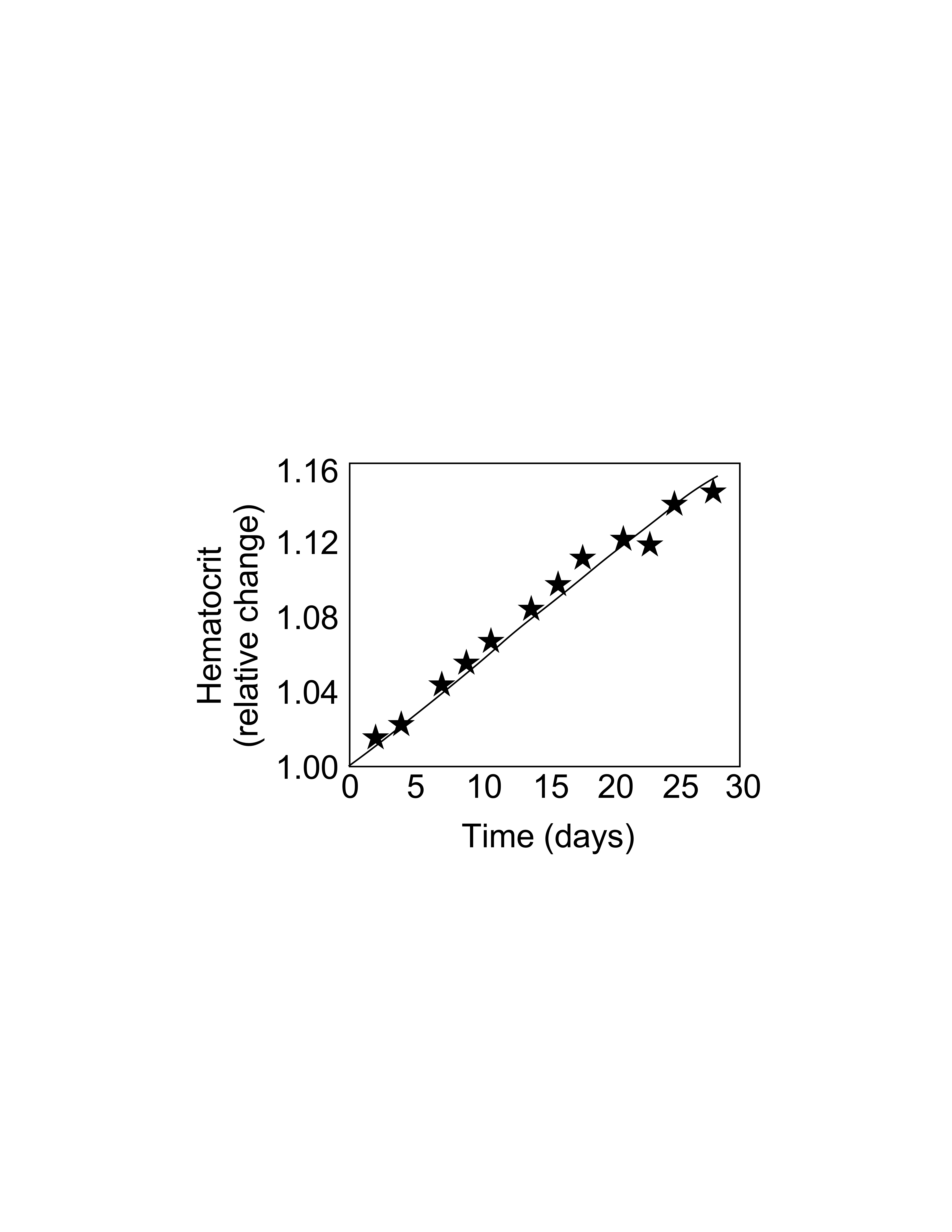

Supplement: S5 Fig — (TIF) [file pcbi.1006060.s005.tif]

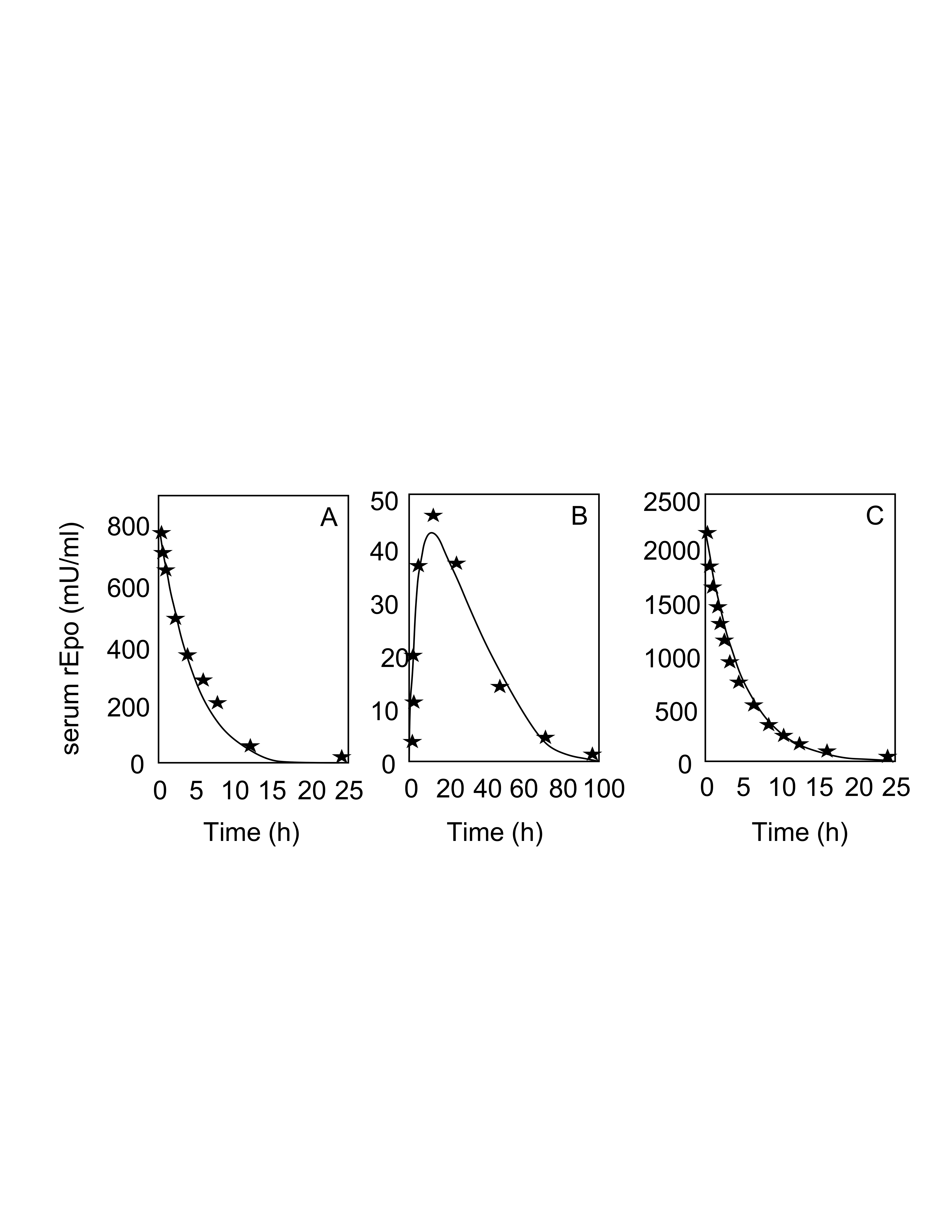

Supplement: S6 Fig — Comparison of model simulation (solid line) against experimental data (*) of serum concentration over time of rEpo with different doses of rEpo injections (A) IV 50IU/Kg (B) 50 IU/Kg SC and (C) 100 IU/kg IV. (TIF) [file pcbi.1006060.s006.tif]

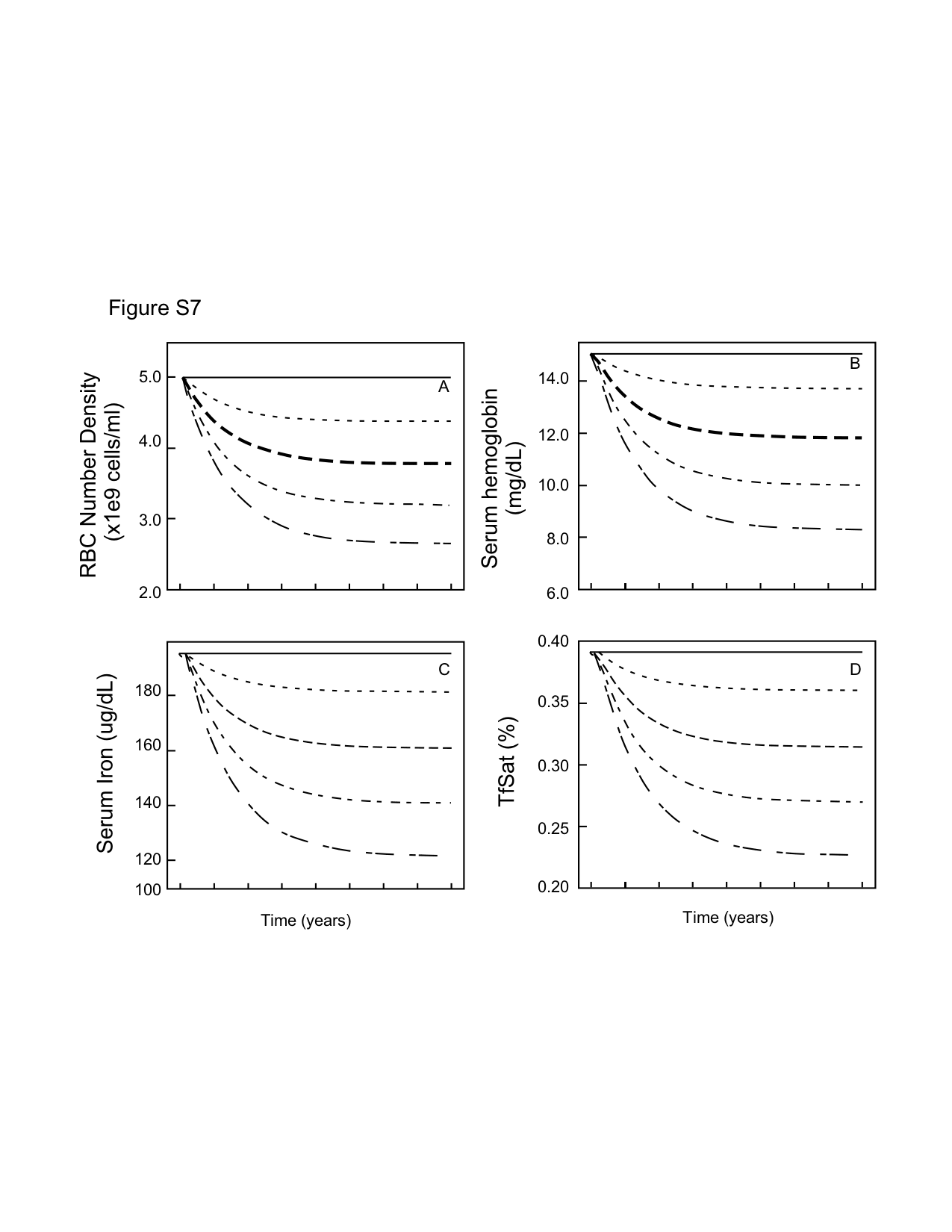

Supplement: S7 Fig — Serum erythropoietin is set to decrease from 6pM to 3.6pM at increments of 0.6pM to represent increasing severity of CKD and the model outputs are shown for a period of 2 yrs. The model can reproduce varying degrees of anemia of CKD as shown with the reduction in plasma red blood cell density (A), plasma hemoglobin (B), serum iron (C) and transferrin saturation (D). (TIF) [file pcbi.1006060.s007.tif]
