# Supplementary material for: Whole-body iron transport and metabolism: Mechanistic, multi-scale model to improve treatment of anemia in chronic kidney disease
Source: PLoS Comput Biol. 2018 Apr 16;14(4):e1006060. doi: 10.1371/journal.pcbi.1006060 (PMC5919696; doi:10.1371/journal.pcbi.1006060)
Supplement: S1 Data — (GZ) [file pcbi.1006060.s008.tar.gz › python_code_submission/readme.rtf]

Input file: Iron_metabolism_parameter_input_file.xlsxright hand side of all odes and some additional function: modules.pyplotting function: plotting.pyDifferential evolution: differential_evolution_map.py1. Steady state calibration for human: steady_state_fit_h.py2. Steady state calibration for mouse: steady_state_fit_m.py3. Phlebotomy experiment: phlebotomoy_fit.py4. Mouse rhHepc injection: mouse_rhHepc_fit.py5. Iron ingestion experiment: iron_ingestion_exp.py6. rEpo 2 compartment PK: rEpo_PK_2compartment.py7. rEpo Pharmcodynamics model: rEpo_PD.py8. Simulation of CKD: CKD_fit.py9. Simulation of CKD with treatment: CKD_rEpo_Fe.pyWithin most routines one can both run calibration and simulation by simply commenting or uncommenting the correct routineAll software requires Python 3.5 with necessary libraries like numpy, scipy etc. Preferred use is with installation of Anaconda for Python 3.5
